# Lipidomic risk scores are independent of polygenic risk scores and can predict incidence of diabetes and cardiovascular disease in a large population cohort

**DOI:** 10.1371/journal.pbio.3001561

**Published:** 2022-03-03

**Authors:** Chris Lauber, Mathias J. Gerl, Christian Klose, Filip Ottosson, Olle Melander, Kai Simons

**Affiliations:** 1 Lipotype GmbH, Dresden, Germany; 2 TWINCORE, Centre for Experimental and Clinical Infection Research, a joint venture between the Hanover Medical School and the Helmholtz Centre for Infection Research, Institute for Experimental Virology, Hanover, Germany; 3 Department of Clinical Sciences, Lund University, Malmö, Sweden; Duke University, UNITED STATES

## Abstract

Type 2 diabetes (T2D) and cardiovascular disease (CVD) represent significant disease burdens for most societies and susceptibility to these diseases is strongly influenced by diet and lifestyle. Physiological changes associated with T2D or CVD, such has high blood pressure and cholesterol and glucose levels in the blood, are often apparent prior to disease incidence. Here we integrated genetics, lipidomics, and standard clinical diagnostics to assess future T2D and CVD risk for 4,067 participants from a large prospective population-based cohort, the Malmö Diet and Cancer-Cardiovascular Cohort. By training Ridge regression-based machine learning models on the measurements obtained at baseline when the individuals were healthy, we computed several risk scores for T2D and CVD incidence during up to 23 years of follow-up. We used these scores to stratify the participants into risk groups and found that a lipidomics risk score based on the quantification of 184 plasma lipid concentrations resulted in a 168% and 84% increase of the incidence rate in the highest risk group and a 77% and 53% decrease of the incidence rate in lowest risk group for T2D and CVD, respectively, compared to the average case rates of 13.8% and 22.0%. Notably, lipidomic risk correlated only marginally with polygenic risk, indicating that the lipidome and genetic variants may constitute largely independent risk factors for T2D and CVD. Risk stratification was further improved by adding standard clinical variables to the model, resulting in a case rate of 51.0% and 53.3% in the highest risk group for T2D and CVD, respectively. The participants in the highest risk group showed significantly altered lipidome compositions affecting 167 and 157 lipid species for T2D and CVD, respectively. Our results demonstrated that a subset of individuals at high risk for developing T2D or CVD can be identified years before disease incidence. The lipidomic risk, which is derived from only one single mass spectrometric measurement that is cheap and fast, is informative and could extend traditional risk assessment based on clinical assays.

## Abstract 2

Risk score analysis of genetic and lipidomic data from a large population cohort reveals that in a subset of patients large-scale alterations in lipidome composition may be prognostic of future type 2 diabetes and cardiovascular disease.

## Introduction

As reported regularly by the World Health Organization (WHO), cardiovascular diseases (CVDs) and diabetes mellitus are among the 10 leading causes of death globally. The risk of developing CVD and type 2 diabetes (T2D) is associated with diet and other lifestyle-related behavior. Therefore, early and accurate identification of individuals at high disease risk is crucial for lowering the disease burden by suggesting tailored and timely countermeasures, for instance, changes to the diet. Risk prediction based on machine learning models has been shown to benefit from the inclusion of omics-based measurements in addition to the classical risk factors such as cholesterol and glucose levels in the blood [1–3]. In particular, assessing variations in the genome, proteome, and metabolome including the lipidome may offer opportunities to identify pathophysiological processes and pathways, which may differ between patients or patient subgroups [4].

Large population-based genotyping efforts undertaken during recent years have demonstrated that many phenotypes, including predisposition to human diseases, are polygenic, i.e., result from a large number of genetic loci, each having a small effect [5,6]. In typical genome-wide association studies (GWAS), these effect sizes are estimated separately for each variant position (usually via regression analysis) because a joint estimation is computationally intractable. After reducing sets of highly correlating variants (for instance, those in strong linkage disequilibrium) to single index variants via pruning or clumping, a summation of the number of risk alleles present in an individual, weighted by their effect sizes, can then be used to quantify the risk of that person for developing the disease considered relative to the average risk in the population.

Although the omics sciences are continuously expanding, their potential for diagnostics and healthcare is not yet fully utilized. Genomics remains by far the leader with more than 80,000 papers published in 2019 on PubMed. Proteomics is number 2 with 12,691 articles, followed by metabolomics with 7,455. Lipidomics, as a separate subbranch of metabolomics, is the latecomer with 1,188 papers. Nevertheless, lipidomics is catching up and has the advantage over proteomics and general metabolomics that affordable internal standards were introduced by LIPID MAPS and made available commercially by Avanti [7,8]. In order to introduce omics technologies in diagnostics and healthcare, quantitation and reproducibility have to be guaranteed. This is difficult to achieve without adding standards to each sample analyzed. We have established a shotgun mass spectrometric platform for lipidomics analysis that performs at high-throughput reproducibly and quantitatively with high precision [9]. Recent research has demonstrated that lipids are sensitive metabolic indicators of change in health and disease [10–15] and also systems biology approaches are improving [16]. Most encouraging is that the blood plasma lipidome seems to reflect the metabolic status in the body and plasma offers an easily accessible resource for lipidomic analysis [1,17–19].

Longitudinal studies assessing associations between the plasma lipidome and risk of developing T2D or CVD have emerged [20,21]. However, comparisons with genetic and other omics-based risk predictions are rarely made, and studies including risk prediction of large population-based cohorts with many incident cases and long follow-up are lacking. In this paper, we have used the Malmö Diet and Cancer-Cardiovascular Study (MDC-CC) with a cohort comprising 4,067 participants that were recruited from 1991 to 1994 and followed until 2015. Our work is a continuation of two previous studies, where we identified a lifestyle lipid profile that predicted T2D and coronary artery disease (CAD) development beyond classical risk factors [2,22]. These previous studies were primarily concerned with the overall performance gain of adding the lipidome on top of current risk factors. Here we are using the same cohort to analyze the lipidomic and polygenic risk in isolation and in combination with each other and with the classical risk factors based on clinical measurements. We apply a supervised machine learning approach to specifically focus on subsets of individuals with highest risk, and we complement this with an unsupervised analysis to stratify the individuals into categories reflecting the risk of future T2D or CVD incidence. For both diseases, we observe a subgroup of individuals with extraordinarily high risk scores and demonstrate that these subgroups are associated with large-scale alternations of the lipidome that may be prognostic for future disease incidence.

## Results

### The Malmö cohort and associated clinical and omics measurements

The MDC-CC is a prospective population-based cohort of participants from Malmö, Sweden, which have been recruited between 1991 and 1994. All participants underwent medical and physical examination and laboratory assessment at baseline. For 4,067 participants, we measured the lipidomes of fasted blood plasma samples taken at baseline by shotgun lipidomics, which involved the determination of molar concentrations of 184 lipid species or subspecies (see Materials and methods for details). A more detailed description of the MDC-CC can be found in a previous paper [2].

The MDC-CC participants have been followed until 2015, and major disease incidences like T2D, CVDs, dementia and different types of cancer, and also cause of death have been recorded. Here, we focus on two diseases in which lipid metabolism is clearly implicated, T2D and CVD, with the aim to assess future incidence risk. For T2D, we excluded all participants with prevalent diabetes at baseline, leaving **3,688** individuals for analysis from whom **509** (**13.8%**) developed T2D during the follow-up period. In the case of the CVD analysis, we excluded all participants that have had a CAD or stroke event prior to baseline. For the **3,951** remaining individuals, we defined **870** (**22.0%**) future incidence cases to be those that had a CAD or stroke event or who died due to a cardiovascular event during follow-up.

### Prospective disease risk quantification by predictive modeling

In order to explore whether disease risk is reflected by changes of the lipidome and other parameters, we built predictive classification models of future T2D and CVD incidences (Table 1). These models form the basis to derive different risk scores reflecting a certain type of variables (for instance, lipid species amounts) or combinations of variable types (for instance, lipid species amounts and clinical measurements). We then stratified both the future incidence cases and the controls into subgroups based on a ranking by their risk scores and analyzed the enrichment of cases in each subgroup relative to the average case rate in the cohort.

**Table 1:** AUC classification metric for the models underlying the different risk scores for T2D and CVD. We used class prediction probabilities averaged over the 10 independent cross-validation iterations during the AUC calculation.

model	T2D	CVD
N	0.533	0.517
P	0.609	0.507
L	0.728	0.661
N + L + P	0.745	0.634
N + L + P + C	0.797	0.659

AUC, area under the curve; CVD, cardiovascular disease; T2D, type 2 diabetes.

#### A null model as baseline

To estimate a “null risk” for developing T2D or CVD, we started with a simple model that only considered age and sex of a person. We then analyzed for both diseases whether this risk score (N) correlated with the case rate in such a way that higher risk score quantiles were enriched with cases compared to the average incidence rates of 13.8% and 22.0% or T2D and CVD, respectively.

**Fig 1:**
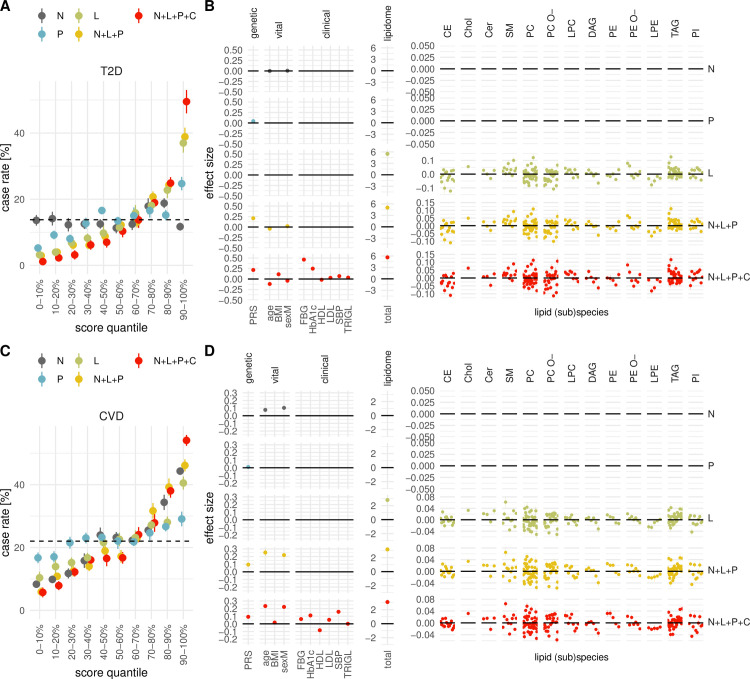
Correlation of different risk scores with future disease incidence rate. Different risk scores are sorted and partitioned into deciles; for each decile, the fraction of future T2D (**A**) and CVD (**C**) incidences is shown, with points indicating the mean over 10 independent repetitions and the bars the associated standard errors of the mean. The risk scores differ with respect to the included predictor variables: null model (N), lipidome (L), polygenic score (P), 7 standard clinical or vital measures (C). Different predictor types may be combined to derive composite scores like N + L + P comprising the null model, lipidome and polygenic predictors. The horizontal dashed line shows the average incidence rate across the full cohort. The effect sizes of individual predictor variables from each model are shown for T2D (**B**) and CVD (**D**) grouped by lipid class or predictor type. The data underlying this figure may be found in S1 Data. BMI, body mass index, sum of absolute effect sizes across all lipid species/subspecies (total); CE, cholesteryl ester; Cer, ceramide; Chol, cholesterol; CVD, cardiovascular disease; DAG, diacylglyceride; FBG, fasting blood glucose; HbA1c, glycated hemoglobin; HDL, high-density lipoprotein; LDL, low-density lipoprotein; LPC, lysophosphatidylcholine; LPE, lysophosphatidylethanolamine; PC, phosphatidylcholine; PC O-, ether- phosphatidylcholine; PE, phosphatidylethanolamine; PE O-, ether- phosphatidylethanolamine; PI, phosphatidylinositol; PRS, polygenic risk score; SBP, systolic blood pressure; SM, sphingomyelin; TAG, triacylglyceride; TRIGL, triglyceride; T2D, type 2 diabetes.

For T2D, no consistent increase in case rate with increasing risk score quantile was observed (**Fig 1A**). In the case of CVD, however, the 80% to 90% and 90% to 100% quantiles showed a case rate of **34.4**% and **44.3**%, respectively. This differed considerably from the average incidence rate as well as from the 0% to 10% and 10% to 20% quantiles, which had a case rate of **6.3**% and **9.8**%, respectively (**Fig 1C**).

#### Quantifying lipidomic risk

We then analyzed whether differences in lipidome composition can modulate the risk for future T2D and CVD. To this end, we included in total 184 lipid species or subspecies concentrations and computed a lipidomics risk score (L). This score showed a clear trend of increase with increasing T2D and CVD incidence rate. In the case of T2D, the lowest 0% to 10% and the highest 90% to 100% quantiles showed highly contrasting case rates of **3.2**% and **37.0**% (**Fig 1A**), corresponding to a 76.8% decrease and 168.1% increase and to an odds ratio (OR) of 0.21 and 3.67, respectively, compared to the average T2D incidence rate (**Fig 1A**). This contrast between the lowest and highest lipidomics risk scores was also observed, although to a lesser extent, for CVD, showing a 52.8% decrease (**10.4**% case rate; OR of 0.41) and a 84.2% increase (**40.5**%; 2.41) compared to the average incidence rate for the 0% to 10% and the 90% to 100% quantile, respectively (**Fig 1C**). The effect sizes of individual lipid species, which correspond to the estimated coefficients in the trained model, varied considerably and were in the range from **−0.125** to **0.125** and **−0.05** to **0.05** for T2D and CVD, respectively. When correlating the L score with lifestyle variables such as a diet risk score [23] and a “health conscious” food pattern [24], we found only marginal effects for both diseases.

As the lipidomics model was outperformed by the null model for CVD (**Fig 1C**), we also computed and compared N + C and N + L + C models and found that addition of the lipidomics features improves the risk prediction with an area under the curve (AUC) of 0.659 compared to 0.607 for, respectively, N + L + C and N + C (S1 **Fig**). These results showed that the lipidome has added value for CVD risk prediction and does not merely capture age- and sex-related lipidome patterns.

**Fig 2:**
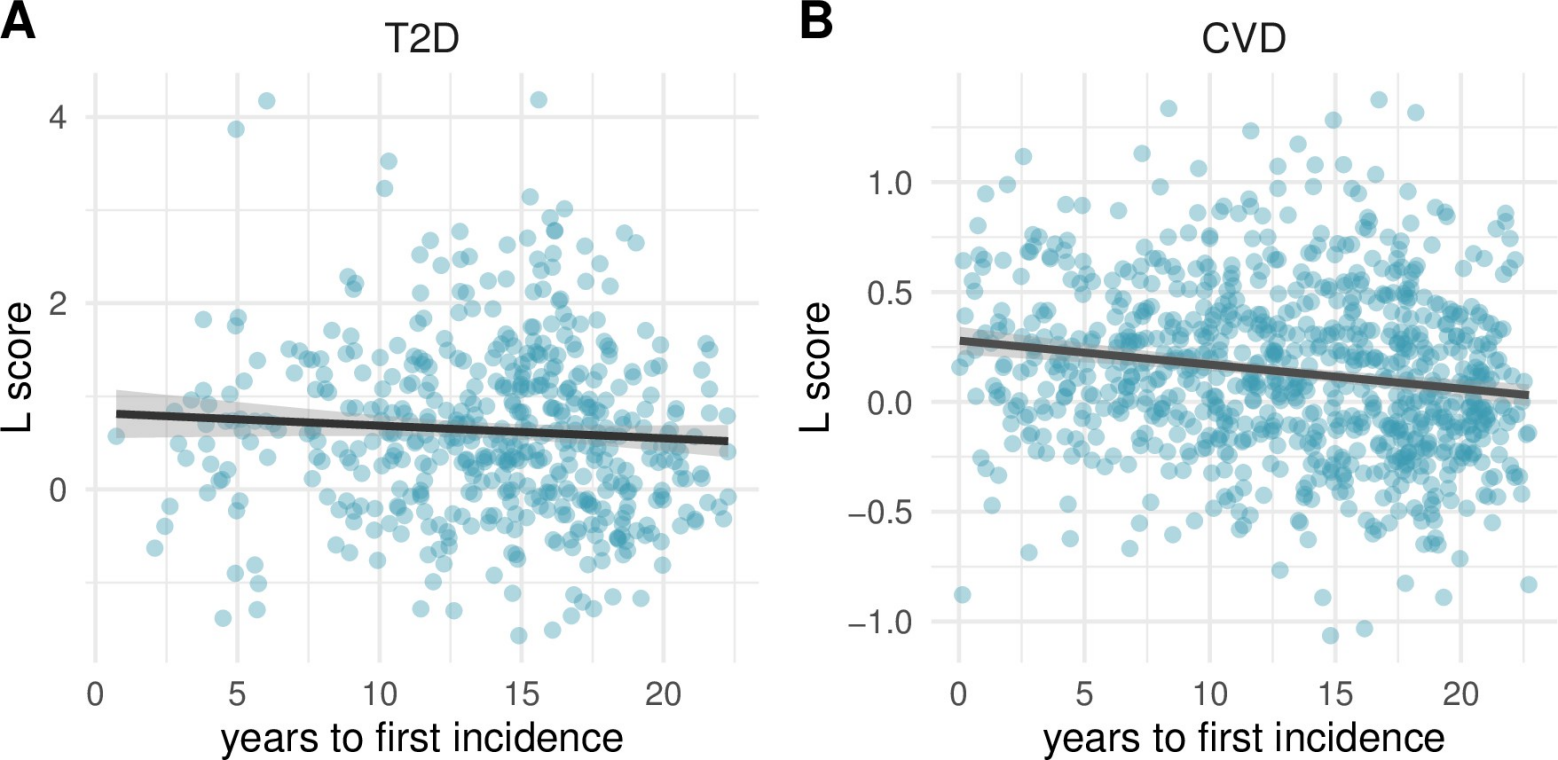
Lipidomic risk score versus time to first incidence. The time in years to the first incidence event is compared to the L risk score for T2D (A) and CVD (B). Pearson’s *r* = −0.107 and −0.169 for T2D and CVD, respectively. The risk scores are average values from 10 independent replications. The curve shows a least squares fit of a linear model to the data. The data underlying this figure may be found in S1 Data. CVD, cardiovascular disease; T2D, type 2 diabetes.

Notably, the L risk score correlated only marginally with time to incidence event for T2D (**Fig 2A**; Pearson correlation, r = **−0.107**, *p* = **0.016**). The negative correlation with time to incidence was stronger for CVD (**Fig 2B**; r = **−0.169**, *p* = **7.8 × 10**^−7^) (**Table 2**). This slight decrease of the L risk with time can be partially explained by the unbalanced age distribution of the analyzed cohort being composed of individuals older than 46 years at baseline. Importantly, individuals with very high lipidomic risk scores were observed across the full timescale for both diseases (**Fig 2**), suggesting that pathological lipidome changes can become established many years before onset of these major diseases. Notably, the negative correlation with time to incidence was much and modestly stronger for a risk score computed only on clinical parameters (C score) for T2D (r = **−0.299**, *p* = **9.3 × 10**^−12^) and CVD (r = **−0.23**, *p* = **1.2 × 10**^−11^), respectively **(S2 Fig, Table 2**), indicating that the C score is of biggest value for predicting shorter term risk.

**Table 2:** Correlation test results of L and C risk scores with time to T2D or CVD incidence. Test statistic (t), *p*-value (p), Pearson’s product–moment correlation coefficient (*r*) and its 95% confidence interval (CI_95%_), and the number of observations (n) are shown.

disease	model	t	p	*r*	CI_95%_	n
T2D	L	−2.41	0.016	−0.107	[−0.193, −0.020]	500
T2D	C	−6.98	9.3 × 10^−12^	−0.299	[−0.376, −0.217]	500
CVD	L	−4.98	7.8 × 10^−07^	−0.169	[−0.233, −0.102]	850
CVD	C	−6.88	1.2 × 10^−11^	−0.230	[−0.293, −0.165]	850

CVD, cardiovascular disease; T2D, type 2 diabetes.

#### Quantifying polygenic risk and assessing its overlap with lipidomic risk

We next wanted to assess whether genetic predispositions have an impact on T2D and CVD risk. In order to do so, we computed a polygenic risk score for each disease, utilizing summary statistics from published GWAS to estimate the effect sizes of individuals’ genetic variants. For T2D, we used data from the DIAGRAM consortium that analyzed 900,000 European-descent individuals from which 9% were cases [5]. This data set contained 23 million single nucleotide variants (SNVs), and 19,214 of them were significantly associated with T2D at the genome-wide level (*p* < 5 × 10^−8^) and were considered for the genetic risk score computation. In the case of CVD, we made use of a meta-analysis uniting UK BioBank and CARDIoGRAMplusC4D data, which comprised in total 150,000 European- and Asian-descent individuals with a case rate of 8% [6]. This data set included 9 million SNVs from which 2,686 variants showed genome-wide significance of CVD association.

Although this polygenic score (P) correlated with T2D incidence rate (**5.2**% versus **24.7**% for the lowest and highest risk score quantile, respectively), its predictive performance was considerably lower than that of the lipidomic (L) score (**Fig 1A**). This relatively minor impact of the polygenic predictors was also reflected by a composite model uniting the null model, lipidomic and polygenic predictors (N + L + P) that resulted only in a marginal improvement compared to the L model (**Fig 1A**). In the case of CVD, the enrichment with incidence cases obtained via the P score was comparably small (**16.7**% versus **29.1**% for the first and 10th quantile, respectively), and the improvement of predictive performance from the L to N + L + P score was mainly driven by the null model parameters (**Fig 1C**).

**Fig 3:**
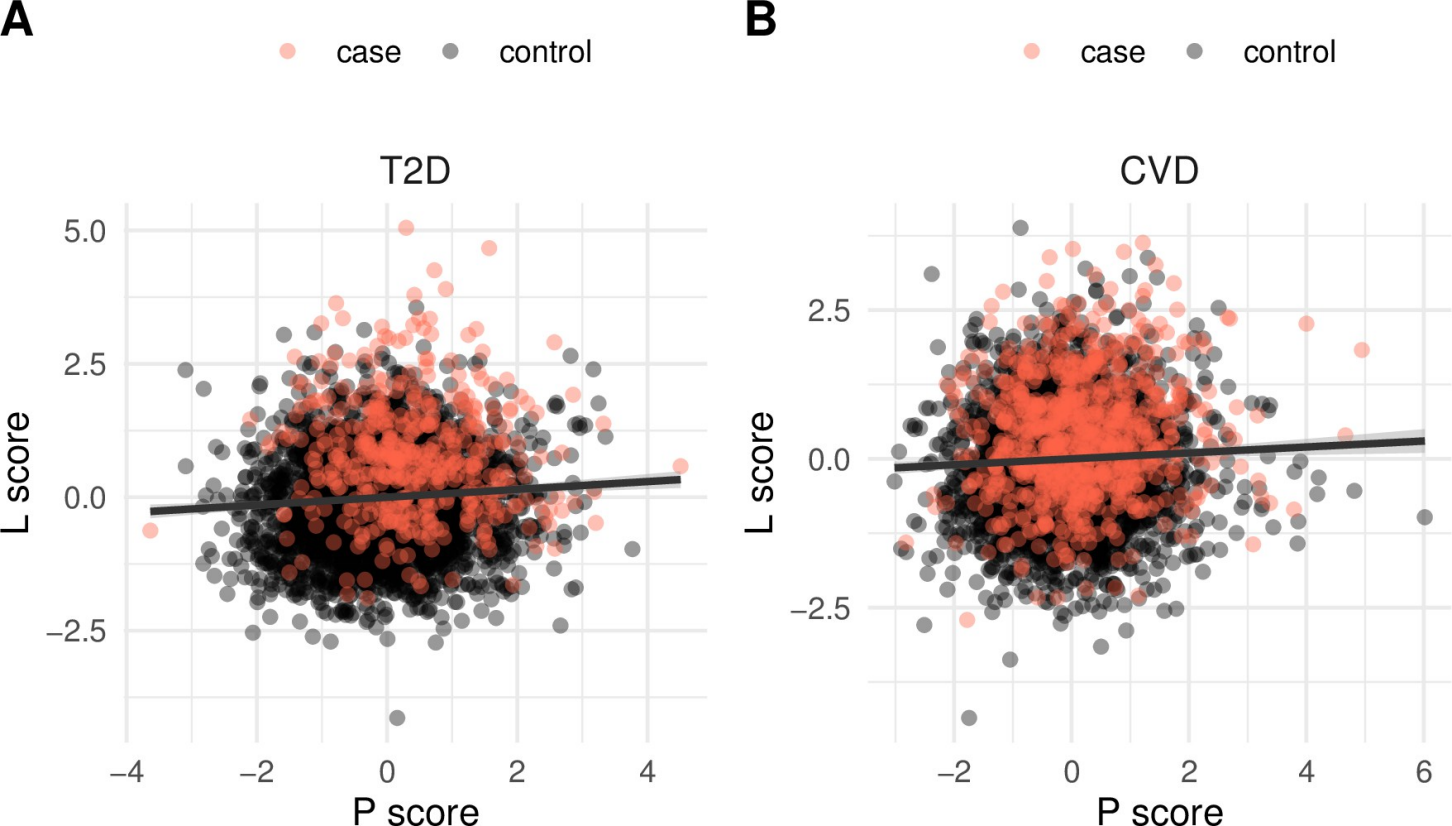
Lipidomic versus polygenic risk. The lipidomic risk (L score) is compared to the polygenic risk (P score) for T2D (A) and CVD (B). Pearson’s *r* = 0.087 and 0.045 for T2D and CVD, respectively. The risk scores are average values from 10 independent replications. The curve shows a least squares fit of a linear model to the data. The data underlying this figure may be found in S1 Data. CVD, cardiovascular disease; T2D, type 2 diabetes.

In order to assess the overlap of the individual contributions of lipidome and genotype to disease risk, we compared these two omics-specific scores and found that they correlated only slightly for both T2D (**Fig 3A**; Pearson’s ***r* = 0.087**, *p* = **4.4 × 10**^−7^) and CVD (**Fig 3B**; *r* = **0.045**, *p* = **0.007**). Specifically, we observed several participants with average genetic risk but high lipidomic risk and vice versa (**Fig 3**), indicating that the lipidome and genotype may constitute largely independent factors of future T2D and CVD incidence risk.

#### Further improving risk stratification by inclusion of standard clinical and vital measurements

To see if risk stratification can be improved further for the two diseases, we extended the model by standard vital and clinical measurements. Specifically, we included body mass index (BMI), systolic blood pressure (SBP), fasting blood glucose (FBG), glycated hemoglobin (HbA1c), low-density lipoprotein (LDL), high-density lipoprotein (HDL), and triglyceride levels as predictor variables. These were combined with polygenic, lipidomic, and null model parameters to derive a composite model. The resulting risk score (N + L + P + C) indeed improved risk stratification compared to the other scores, in particular and disproportionally for the highest risk score quantile (**Fig 1AC**).

For T2D, the 90% to 100% quantile showed a case rate of **51.0%**, corresponding to a 258.8% increase (OR of 6.12) compared to the average incidence rate. Notably, the case rate for the lowest 0% to 10% quantile was only **1.1%** (OR of 0.07) (**Fig 1A**), corresponding to just **4** out of **369** individuals in this quantile-based subgroup when extrapolating to the full cohort size of **3,688** considered in the T2D analysis. The highest effect sizes were attributed to FBG and HbA1c (**Fig 1B, S1 Table**), which is not surprising as these measurements quantify the short-term and long-term blood sugar levels, respectively. Also, the genetic score showed a rather high effect size comparable to that of HbA1c, while the effect sizes of the other standard parameters including BMI as well as those of individual lipid species were small. However, when combining the absolute effect sizes of all lipid species, their total effect of **5.33** exceeded that of any other predictor (**Fig 1B, S1 Table**).

In the case of CVD, a case rate of **53.3%** in the highest 90% to 100% quantile corresponded to a 145.9% increase (OR of 4.17) relative to the average incidence rate (**Fig 1C**). The case rate in the lowest 0% to 10% quantile was **6.5%** (OR of 0.22), corresponding to 26 out of 395 individuals, considerably more than observed for T2D in this lowest-risk quantile. Age, gender, and SBP showed the highest effect on CVD risk (**Fig 1D, S1 Table**). The effect size of the genetic score was moderate but considerably lower than in the T2D analysis (**Fig 1BD**). As for T2D, the combined effect size of all lipid species of **3.31** exceeded those of other individual predictors also for CVD risk (**Fig 1D, S1 Table**).

We then analyzed the overlap of high-risk T2D and CVD by considering the **3,445** participants for which the N + L + P + C risk score has been calculated for both diseases. From the 345 individuals in the 90% to 100% risk score quantile there were only **35** individuals (**10.1%**) that were assigned the highest risk for both T2D and CVD, while the remaining 89.9% showed varying risk for the other disease (S3 **Fig**), demonstrating that only a relatively small subset of individuals from the MDC-CC cohort had the highest risk for developing both diseases analyzed here.

### A high-risk lipidome signature

**Fig 4:**
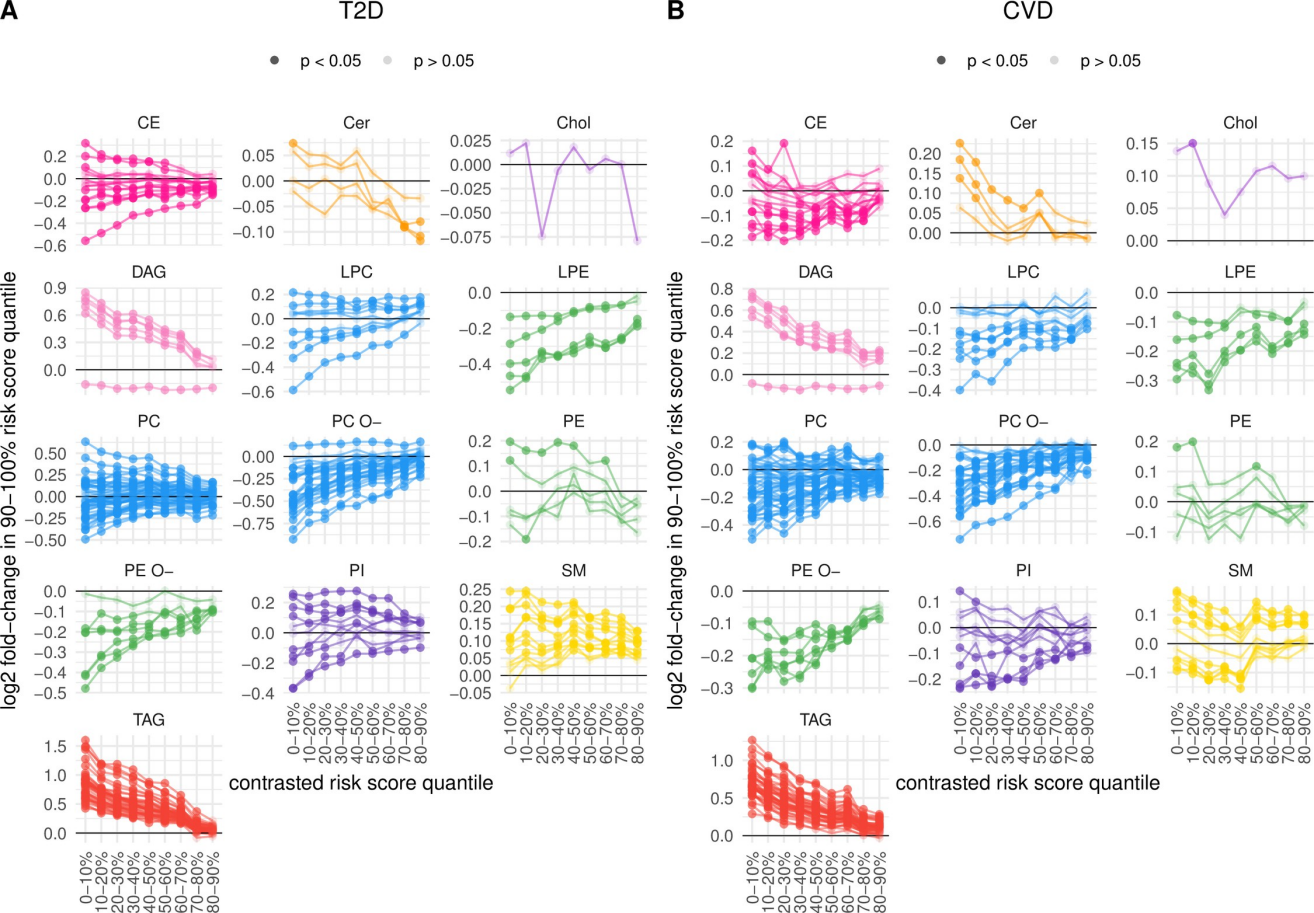
High-risk lipidome signatures. Shown are fold-changes of lipid concentrations in the individuals of the 90%–100% N + L + P + C risk score quantile relative to those of each of the other 9 quantiles for T2D (A) and CVD (B). Fold-change values of separate quantile comparisons per species are shown by points, which have high opacity if the difference of mean lipid concentration was statistically significant (after correction for multiple testing) according to a *t* test; otherwise, points are drawn with high transparency for nonsignificant changes. Positive and negative values correspond to, respectively, increased and decreased concentrations in the high-risk 90%–100% quantile participants, while the horizontal line at zero indicates no change. Points corresponding to the same lipid species are connected by a line. See also S2 Table for species names. The data underlying this figure may be found in S1 Data. CE, cholesteryl ester; Cer, ceramide; Chol, cholesterol; CVD, cardiovascular disease; DAG, diacylglyceride; FBG, fasting blood glucose; HbA1c, glycated hemoglobin; HDL, high-density lipoprotein; LDL, low-density lipoprotein; LPC, lysophosphatidylcholine; LPE, lysophosphatidylethanolamine; PC, phosphatidylcholine; PC O-, ether- phosphatidylcholine; PE, phosphatidylethanolamine; PE O-, ether- phosphatidylethanolamine; PI, phosphatidylinositol; PRS, polygenic risk score; SBP, systolic blood pressure; SM, sphingomyelin; TAG, triacylglyceride; TRIGL, triglyceride; T2D, type 2 diabetes.

To identify constituents of the lipidome that are altered specifically in the highest risk individuals, we conducted statistical comparisons of lipid concentrations between participants of the 90% to 100% N + L + P + C risk score quantile and those of the other nine quantiles. For T2D, **91** lipid species (**49.5%**) showed a significantly higher concentration in the highest-risk individuals in at least one of the nine comparisons while another **76** (**41.3%**) species had significantly decreased concentrations (**Fig 4A, S2 Table**). The remaining **18** (**9.8%**) species were not altered. For most of the altered lipids, there was a clear correlation of concentration fold-change with risk score difference. Specifically, the change of lipid concentrations in individuals of the 90% to100% quantile was highest when compared to the individuals in the 0% to 10% quantile and this difference decreased toward the 80% to 90% quantile (**Fig 4A**). This trend was most pronounced for all TAG species and all except one DAG species, showing elevated concentrations in the highest risk individuals, as well as for most PC O- species showing decreased concentrations. Notable exceptions included DAG 18:1;0_18:3;0, which was consistently decreased in concentration in the highest risk individuals with similar fold-change in all quantile comparisons. Likewise, the concentration of PC O-16:1;0/20:3;0 represented the only PC species that was consistently elevated in the individuals from the 90% to 100% quantile compared to all others. Moreover, most sphingomyelin (SM) species in the high-risk individuals showed a significant increase of concentration that did not strongly correlate with risk score difference (**Fig 4A**).

In the case of CVD, **67 (36.4%)** lipid species showed a significant increase in concentration in the highest-risk individuals in at least one comparison while another **90** (**48.9%**) species had decreased concentrations. There were striking similarities between the high-risk CVD and T2D signatures (**Fig 4AB, S2 Table**). These included the significantly elevated TAG and DAG levels and also the consistent decrease of DAG 18:1;0_18:3;0 concentration in the high-risk individuals. Patterns of altered PC, LPE, and PE O- species were comparable between the two diseases, too. However, there were also differences in the lipidome signatures between high-risk CVD and T2D. PC O-16:1;0/20:3;0 concentration was not altered for CVD (**Fig 4B**). There was no clear pattern of elevated PI levels in high-risk CVD individuals, compared to roughly half of PI species with increased concentrations for T2D; a similar difference was observed for LPC species. Likewise, only a subset of SM species showed statistically increased concentration in the high-risk CVD individuals compared to an effect on all SM species in T2D. Moreover, three Cer species showed statistically elevated levels in the high-risk CVD individuals when contrasted with low-risk participants (**Fig 4B**), while no consistent Cer alterations were apparent for T2D (**Fig 4A**).

We extended our analysis of differential lipidome alterations in the individuals of the 90% to 100% quantile to the hydrocarbon chain (HC; fatty acid and fatty alcohol) levels within complex lipids. After summing up HC concentrations across lipid classes, we observed striking differences in concentration fold-change patterns between the 18 analyzed HCs for both diseases (S4 **Fig**). The alkyl chains (FOH) all showed decreased concentrations in the participants of the 90% to 100% quantile, suggesting a protective effect. In contrast, some of the acyl chains (FA) had increased concentrations in these high-risk participants, while others were unchanged. We did not identify correlations of the strength of FA concentration fold-change with the saturation level of a FA (number of double bonds) but noticed that many of the risk-associated FAs have rather short chain lengths, for instance, 14:00;0, 16:00;0, 16:1;0, 17:1;0, and 18:1;0 (S4A and S5B **Fig**).

### Clustering the lipidomes into subgroups that correlate with disease risk

To further explore how distinct changes of the lipidome composition may correspond to different risk subgroups for developing T2D or CVD, we constructed a clustering of the participants based only on the lipidomics data. We excluded both prevalent T2D and prevalent CVD cases in this analysis, leaving **3,599** participants. Notably, this unsupervised analysis only considered the dissimilarity of lipidome pairs and did not make any use of health/disease status or other clinical or personal information. It is therefore complementary to the supervised risk score analysis.

**Fig 5:**
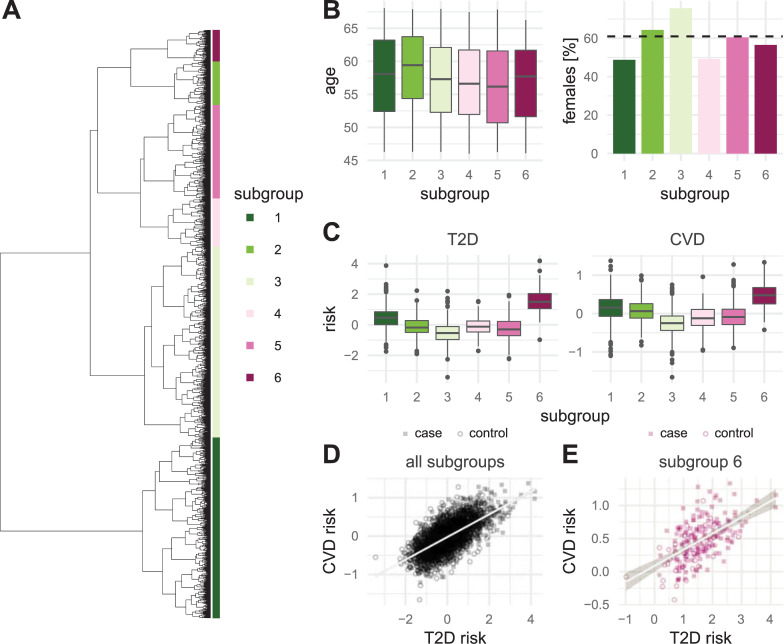
Lipidome-based risk subgroups. Shown is a hierarchical clustering of 3,599 plasma samples that is solely based on 184 lipid concentrations. Pearson correlation has been used as distance measure. The tips of the dendrogram are colored according to the 6 subgroups obtained by splitting the dendrogram into the 6 most basal clusters. (B) Distribution of age (left) and sex (right) in the 6 subgroups. The dashed horizontal line indicates the average female percentage in the full cohort. (C) Distribution of L risk scores for the 6 subgroups for T2D (left) and CVD (right). (D) The 2 risk scores for T2D and CVD are compared for all participants; note that cases are defined here as being either a T2D or CVD incidence. The line indicates a least squares fit of a linear model to the data; Pearson’s *r* = 0.613. (E) Same as C but only for participants belonging to subgroup 6; Pearson’s *r* = 0.504. The data underlying this figure may be found in S1 Data. CVD, cardiovascular disease; T2D, type 2 diabetes.

According to the clustering dendrogram, we could cluster the participants into six major subgroups that contained from **183** to **949** individuals, which had varying risk score distributions (**Fig 5A**). No major differences in age or sex distributions between the subgroups were apparent (**Fig 5B**). While some of the subgroups had low or average risk scores, subgroup 6 clearly stood out by showing considerably higher risk scores than the other five subgroups for both T2D and CVD (**Fig 5B**). The relations between the subgroups in the dendrogram did not correlate with the average risk scores of the subgroups. Subgroup 6, for instance, clustered far away from subgroup 1 that ranked second by average risk score. Notably, the risk score profiles across the different clusters were strikingly similar between T2D and CVD, also shown by a strong correlation of 0.613 between the two L scores (**Fig 5D**). This correlation remained when looking exclusively at subgroup 6, which was strongly enriched with future incidence cases as **145** of the **183** individuals in this subgroup developed T2D or CVD during the follow-up period, corresponding to a case rate of **79.2%** (**Fig 5E**).

When comparing the vital and clinical parameters between participants from subgroup 6 and those from the other subgroups, we did not find statistically significant differences with the exceptions of FBG, which was slightly increased, and HDL, which was slightly decreased in the subgroup 6 individuals (**Table 3**). In particular, neither age nor sex differed significantly. In contrast, 114 lipid species concentrations showed significant differences between subgroup 6 and the other subgroups **(S3 Table**). The top lipid species according to *p*-value was DAG 18:1;0_18:3;0 (log_2_ fold-change subgroup 6 versus other subgroups of **−1.136**), followed by LPE 20:4;0 (**−0.611**) and PC 15:0;0_18:2;0 (**−0.598**). DAG 18:1;0_18:3;0 was already identified as a marker of the highest risk individuals (see above). Notably, from the top 30 lipids ranked by *p*-value all showed decreased concentrations in subgroup 6 relative to the other subgroups, with the sole exception of seven SM species, which had elevated concentrations. The remaining four SM species also showed higher concentration in subgroup 6 individuals compared to the other participants (**S3 Table**). This statistically supported difference of SM concentrations was also reflected at the lipid class level where SM ranked top according to *p*-value (**Table 3**). In total nine lipid classes were significantly altered in subgroup 6 while PC, PC O-, DAG, and TAG did not show significant differences. It is interesting that the increase in SMs is balanced by a decrease of other lipids, many containing more than one double bond, suggesting that the changes have to do with increasing the ordering of the cell membranes (21–23), and this may unbalance the body’s lipidome and predispose for disease. In summary, our results show that pathological alterations of the lipidome may arise years before a disease is diagnosed, therefore offering new opportunities for early risk assessment.

**Table 3:** Comparison of vital, clinical, genetic, and lipidomic variables between participants from subgroup 6 and the other participants. *t* Tests were used except for the fraction of males for which a chi-squared test on the absolute counts was applied. *p*-Values were adjusted for multiple testing; bold font indicates adjusted *p*-values smaller than 0.05.

parameter	type	mean subgroups 1 + 2 + 3 + 4 + 5	mean subgroup 6	log_2_ fold-change	adjusted *p*-value
age	vital	57.4	56.9	−0.013	0.309
male%	vital	0.389	0.435	0.159	0.335
BMI	vital	25.3	25.7	0.021	0.259
SBP	clinical	140.8	142.8	0.021	0.187
HDL	clinical	1.418	1.346	−0.075	**0.007**
LDL	clinical	4.158	4.251	0.032	0.260
TRIGL	clinical	1.253	1.349	0.107	0.064
FBG	clinical	4.898	5.021	0.036	**0.001**
HbA1c	clinical	4.798	4.793	−0.002	0.900
PRS	genetic	−0.178	−0.178	−0.0001	0.402
SM	lipid class	167.3	204.5	0.290	**4.4 × 10** ^−25^
CE	lipid class	5,774.2	4,952.2	−0.222	**1.1 × 10** ^−21^
LPE	lipid class	5.0	4.1	−0.315	**1.2 × 10** ^−21^
PE O-	lipid class	28.5	22.4	−0.350	**2.7 × 10** ^−21^
LPC	lipid class	142.8	170.8	0.259	**5.8 × 10** ^−18^
PE	lipid class	13.8	11.2	−0.299	**1.8 × 10** ^−10^
Cer	lipid class	6.5	5.7	−0.170	**2.1 × 10** ^−10^
Chol	lipid class	961.6	1,104.8	0.200	**2.1 × 10** ^−10^
PI	lipid class	37.6	40.7	0.113	**1.6 × 10** ^−04^
PC	lipid class	1,672.8	1,730.1	0.049	0.058
DAG	lipid class	26.9	28.0	0.055	0.336
PC O-	lipid class	61.8	60.6	−0.029	0.336
TAG	lipid class	1,278.6	1,288.3	0.011	0.836

BMI, body mass index; CE, cholesteryl ester; Cer, ceramide; Chol, cholesterol; DAG, diacylglyceride; FBG, fasting blood glucose; HbA1c, glycated hemoglobin; HDL, high-density lipoprotein; LDL, low-density lipoprotein; LPC, lysophosphatidylcholine; LPE, lysophosphatidylethanolamine; PC, phosphatidylcholine; PC O-, ether- phosphatidylcholine; PE, phosphatidylethanolamine; PE O-, ether- phosphatidylethanolamine; PI, phosphatidylinositol; PRS, polygenic risk score; SBP, systolic blood pressure; SM, sphingomyelin; TAG, triacylglyceride; TRIGL, triglyceride.

## Discussion

### Risk score–based versus classical classification analysis

Our risk score–based approach differs from the more traditional approaches basing on Cox regression models that explicitly account for the time to disease incidence during modeling and which typically assess the overall performance of a model or the performance gain brought by adding parameters to a model [2,3]. A major aim of our study was the stratification of individuals into subgroups with varying risk profiles and the identification of alterations in lipidome composition that are associated with the subgroups of highest risk. For building the models underlying the risk scores, we chose Ridge regression over Cox because Ridge can account, at least partially, for the high degree of multicollinearity in the lipidomics data and is therefore expected to deliver more precise parameter estimates [25]. We acknowledge that we could have done an initial dimensionality reduction and use the resulting orthogonal components as input for the Cox analysis, but we would then lose a lot on the interpretability of the results, in particular regarding the predictor variables and their effect sizes. When computing risk scores based on such a Cox-based analysis, we found that model performance (AUC values and case rates in the 90% to 100% quantile) was even slightly reduced for N, L, N + L + P, and N + L + P + C (S5 **Fig**). Furthermore, excluding information about time to event from the main Ridge-based models provides supporting evidence for our finding that the lipidome is a risk factor independent of time.

Our method of calculating the different risk scores is akin to the canonical approach used in polygenetic risk studies. The only difference during calculation of the lipidomic and composite risk scores compared to the polygenic risk score is that we jointly estimate the effect sizes of all lipids (and clinical variables) considered via a single Ridge regression analysis. The lipidomic risk score therefore emulates the way polygenic risk scores are derived. The fact that we computed polygenic and phenotypic risk in an analogous way, and that both the blood plasma samples used for lipidomics analysis and the clinical measurements were taken at baseline, enabled us to evaluate and compare the predictive and prognostic power of the genetic and phenotypic risk scores.

We acknowledge that the lack of an external validation cohort is a limitation of our study, but we do not consider this to be of major concern because of the large size of the analyzed cohort and the internal cross-validation of all our models.

### Supervised versus unsupervised analysis

We also computed a lipidome-based hierarchical clustering of all the individuals in the Malmö cohort. This unsupervised analysis only considered the similarity, or dissimilarity, of lipidome pairs and did not make any use of other parameters concerning health or disease status. The fact that the class labels (incidence disease versus control in our case) are not considered when deriving subgroups via this approach makes the hierarchical clustering complementary to risk score analysis, which did use the information on future disease incidence during the modeling. Interestingly, we were able to discern six clusters from the hierarchical clustering and one of them, cluster 6, was strongly enriched with participants with high risk scores for both T2D and CVD while the other clusters were composed predominantly of low-risk individuals or a mixture of low-, intermediate-, and high-risk individuals. The good agreement with respect to the high-risk individuals between this unsupervised analysis and the supervised risk score analysis is reinsuring. It demonstrates that alterations in lipidome composition, like those observed for the individuals in cluster 6, can serve as a prognostic risk factor to identify people with high risk for developing T2D or CVD.

### Lipidome versus polygenic versus clinical measurement-based risk

By first considering only the lipidomics measurement to derive a human plasma lipidome-specific risk score, we demonstrated that this score alone has considerable predictive power for assessing future risk of developing T2D and CVD. The Malmö cohort included 3,688 nondiabetic individuals at baseline, and we found that 509 developed T2D during the follow-up period of over 20 years. According to the lipidomic risk score, the estimated incidence rate in the highest risk group comprising 10% of the whole population was 37%. Similarly, for CVD risk, the highest risk group had an incidence rate of 40.5%. In this case, we had 3,951 individuals at baseline that had no prevalent CVD, out of which 809 fell ill with CVD during the follow-up. Notably, we cannot exclude that some of the “controls” in the highest risk group(s) developed the disease after end of the follow-up period. We then assessed the polygenic risk score of all the individuals for the Malmö population cohort from the GWAS data and their association with T2D and CVD. These were lower than the lipidomic risk scores. The polygenic risk for T2D was 24.7% in the highest decile and correspondingly, for CVD, 29.1%. When we compared the lipidomics risk scores with the polygenic risk scores, they correlated only slightly with each other. This was also confirmed by finding that in the highest lipidomics risk score decile for T2D, few high risk score CVD patients were present and vice versa. The risk scores therefore seemed to be highly specific for the two diseases.

Furthermore, we combined the lipidomic and the polygenic risk scores to find out whether this changed the risk estimate. The obtained improvement was small for both T2D and CVD. Although the lipidome and the genotype constituted largely independent factors for future T2D and CVD risk, this seemed not to enhance their predictive power when combined [26]. One reason for this could be that we only analyzed the lipidome once at base line. Because lipidomics is a phenotypic measure that is influenced by lifestyle, one has to consider that the lipidome is not constant but is bound to change with time. This could limit predictive power. Future studies with repeated sampling would be needed to address this. Another reason for the lack of added value of combining P + L could be that P is not a very informative risk score for the two diseases studied and does not add much information beyond that already provided by the L score.

When the predictive model was extended by addition of standard clinical measurements, risk stratification was improved. For T2D risk, the 90% to 100% quantile increased to 51%. In the case of CVD, the case rate rose to 53.3%. Likewise, we obtained a small performance gain when adding the lipidomics features to a standard clinical model, consistent with previous findings [2,3]. Notably, we observed a negative correlation between the extended risk score (considering clinical, genetic, and lipidomic parameters) and time to incidence for both diseases, suggesting that an improvement of risk prediction by inclusion of clinical measurements can most probably be accomplished for individuals who will develop the disease within a rather short period of time after examination. The lipidome-only risk score, in contrast, showed no such time dependence. Overall, it is satisfying that the lipidomic measurement, which is derived from only one single mass spectrometric measurement, is so informative. This is technically a great advantage compared to other risk scores. The method is quantitative, relatively cheap, and fast.

### Switch-like versus gradual establishment of a high-risk lipidome

Two observations suggest that the establishment of a high-risk lipidome might not be gradual but rather switch-like. First, we observed only a very weak correlation between the lipidomic risk score and the time to first disease incidence (Fig 2), in contrast to the predictive power of an alternative risk score based on standard clinical measurements, which showed a trend of decreasing values with increasing time to incidence (S2 Fig). High levels of the lipidomic risk might thus be associated with an individual for years before incident disease occurs. Second, we did not observe a correlation between the distance of subgroups in the lipidome-based clustering analysis and their average lipidomics risk scores (Fig 5). In particular, the two subgroups with the highest average L scores (subgroups 6 and 1) were very distant in the clustering dendrogram. Together, these observations indicate that disease-associated lipidome alterations may appear to occur abruptly if accumulated at a nonlinear, for instance, exponential, rate. One potential factor causing such switch-like behavior could be that a tipping point had been reached where lifestyle caused a breakdown of metabolic homeostasis that in the end would lead to disease [27]. The MDC-CC participants analyzed here had an age distribution of 46 to 68 years at baseline, so it would be interesting to analyze younger longitudinal cohorts in future studies.

Alternatively, the proximity of high- and low-risk subgroups in the clustering and the poor correlation of lipidomic risk with incidence time might suggest that disease architecture differs between patients, in particular between those from different subgroups [4]. This “palette model” proposes that individuals can have multiple defects affecting several, but not all, pathophysiological processes that contribute to disease risk and progression. The underlying causative factors may be genetic and/or environmental, and the potentially distinct paths taken could be reflected by different alterations of lipidome composition, despite the outcome being similar. The establishment of a high-risk lipidome could then still be gradual within an individual patient but may differ substantially between patients.

### The lipidome as a prognostic disease indicator

The lipid species that our shotgun lipidomics platform measures are the lipids that constitute the bulk of the lipids in cellular membranes. Membranes are “hotbeds” of activities and play a significant role in cell and tissue function [28–30]. Many cellular functions take place membrane-bound. It is also becoming clear that the proteins do not perform their functions in cell membranes alone. They require lipid help. Another intriguing feature of cell membranes is their capability to subcompartmentalize into dynamic nanodomains—lipid rafts [31–34]. This concept implies that the overall lipid composition in different cell types is maintained and fine-tuned in each cell membrane at a level to support normal membrane function [35]. But as we cannot sample the lipids in the cell membranes, we are analyzing the lipids in the plasma that are carried around by the plasma lipoproteins. Increasing evidence is demonstrating that the cellular lipidome is changing in pathological states [10,11,14,15,17,20,21]. Indeed, we identified distinct signatures of lipidome alterations in the individuals with highest T2D or CVD risk, which were apparent at both the lipid species and fatty acid level. Moreover, when using the same approach for calculating an L score for future obesity (BMI of 30 or higher) for in total 2,295 MDC-CC individuals with available BMI data at the end of the follow-up period, we (i) found that this score correlated with obesity incidence rate; (ii) observed that this score was largely lipidome-driven; and (iii) identified a sizable fraction of individuals with extreme risk scores that started with normal BMI and became obese during the follow-up period. This suggests that a lipidomics risk score as a prognostic indicator might extend beyond the two diseases studied here, although validation of these preliminary data in a larger and more age-balanced cohort is pending. The utility of lipidomic risk scores could be relevant long before clinical manifestation of disease when a person is still healthy. A useful application could therefore be the monitoring of health (rather than disease) status and how it is affected by lifestyle and diet. This would require measuring of the lipidome at regular intervals, ideally from young to old age, to explore possible links between changes in lifestyle/diet, lipidome alterations, and transitioning into pathological states.

How could the plasma lipids reflect what is going on in the whole body? We hypothesize that plasma lipoproteins are functioning as a surveillance system that helps to maintain the lipid composition in our tissues. The VLDL-LDL lipoproteins are synthesized in the liver, delivering cholesterol to peripheral cells that contain the LDL receptor uptake mechanism in their plasma membranes [36]. The HDL series of lipoproteins is working in the opposite direction and retrieves cholesterol from the periphery for delivery to the liver [37–39]. These lipoproteins are surely not only carrying cholesterol around, but so far, research has been mostly focused on cholesterol. The lipoproteins contain most other lipids found in cell membranes as well, and they could be involved in regulating cellular lipid composition body-wide. They might function as general lipid transporters that play a role in regulating the lipid composition in tissues so that cell membrane function is kept intact. The lipoproteins could indeed constitute extracellular lipid surveillance and delivery vehicles required to regulate metabolic homeostasis and make us to some extent independent of the diet that we consume, at least on short timescales. Lipid metabolism has the potential to be centrally involved in regulating cellular function. If this thesis is validated, lipidomics will be a convenient way to follow body metabolism. Future research will have to find out whether our hypothesis is tenable or not and demonstrate how informative the plasma lipidome is for health and disease.

## Materials and methods

### Study population and endpoints

The Malmö Diet and Cancer Study is a prospective population-based cohort that enrolled 30,447 participants between 1991 and 1996 (participants were born between 1923 and 1950). Baseline information on lifestyle and clinical factors was collected using an extensive questionnaire and clinical examination as previously described [40]. Among these 30,447 participants, a subset of 5,400 participants were randomly selected at enrollment to comprise the MDC-CC and these participants came fasted on a separate occasion for measurement of metabolic variables and storage of serum and plasma in a biobank. For the current study, stored plasma samples for analysis of the lipidome were available in 4,067 individuals.

Weight (kg) and height (m) were measured for assessment of BMI (kg/m^2^). Prevalent diabetes at the baseline examination was defined as follow: (i) a self-reported physician’s diagnosis; (ii) a fasting whole blood glucose concentration of ≥6.1 mmol/L (which equals a plasma glucose level of ≥7.0 mmol/L); or (iii) use of antidiabetic medication. An oscillometric device was used to measure blood pressure (mm Hg) in the supine position twice after 5 minutes of rest. Measurements (mmol/L) of fasting total cholesterol, HDL cholesterol, HbA1c, triglycerides, and glucose were obtained following standard procedures established at the Department of Clinical Chemistry at Malmö University Hospital. The Friedewald equation was used to estimate LDL cholesterol.

The ethics committee at Lund University approved study protocols (DNR 2009/633) and a written informed consent was obtained from all participants.

CVD was defined as either CAD or stroke. Within the Malmö Diet and Cancer Study, CAD was defined as fatal or nonfatal myocardial infarction, coronary artery bypass graft surgery, percutaneous coronary intervention, or death due to CAD. The Swedish Hospital Discharge Register, the Swedish Cause of Death Register, and the Swedish Coronary Angiography and Angioplasty Registry were used to identify CAD cases [41,42]. Myocardial infarction was defined based on either International Classification of Diseases Ninth Revision (ICD-9) code 410 or 10th Revision (ICD-10) code I21. Information about coronary artery bypass surgery was obtained from the national Swedish classification systems of surgical procedures and defined as procedure codes 3065, 3066, 3068, 3080, 3092, 3105, 3127, or 3158 (the Op6 system) or procedure code FN (the KKÅ97 system). Percutaneous coronary intervention status was obtained from the Swedish Coronary Angiography and Angioplasty Registry. Death due to CAD was defined as ICD-9 codes 412 and 414 or ICD-10 codes I22, I23 and I25.

Stroke was defined using codes 430, 431, 434, and 436 (ICD9) and I60, I61, I63, and I64 (ICD10).

New-onset diabetes cases were recruited from 6 different regional and national diabetes registers: This included individuals registered in either of the following: (i) the nationwide Swedish National Diabetes Register; (ii) the regional Diabetes 2000 register of the Scania region [43]; and (iii) the Swedish National Patient register [41]. The latter is a principal source of data for numerous research projects. It covers more than 99% of all psychiatric and somatic hospital discharges as well as Swedish Hospital–based outpatient care. Moreover, individuals were classified as diabetic if they had diabetes as a cause of death according to the Swedish Cause of Death Register, comprising all deaths (occurring in Sweden or abroad) among Swedish residents. Furthermore, individuals were considered diabetic if they had been prescribed antidiabetic drug medication according to the Swedish Prescribed Drug Register [44]. They were also classified as diabetes cases if they had at least 2 HbA1c recordings of ≥6.0% (applying the Swedish Mono-S standardization system). This HbA1C threshold corresponds to ≥7.0% in the US National Glyco hemoglobin 5 Standardization Program (NGSP), in the Malmö HbA_1c_ register [45]. The latter involved analyzing and cataloging all HbA_1c_ samples, obtained both in institutional and noninstitutional care, in the greater Malmö area from 1988 onwards by the Department of Clinical Chemistry. MDC-CC data discussed in the paper will be made available to readers based on a written application to the MDC-CC steering committee (info(at)med.lu.se).

### Lipidomics

#### Lipid extraction for mass spectrometry lipidomics

Mass spectrometry–based lipid analysis was performed as described [9]. Spectra were analyzed with in-house developed lipid identification software based on LipidXplorer [46,47]. An occupational threshold of 70% was applied to the data, keeping lipid species, which were present in at least 70% of the participants. Median coefficient of subspecies variation as accessed by reference samples was 10.49%. For the risk modeling analysis (see below), values (amounts) of a lipid species missing in some individuals were imputed using the median of all values available for that lipid species.

#### Lipid nomenclature

Lipid nomenclature was done as previously described [1]. Specifically, lipid molecules are identified as species or subspecies. Fragmentation of the lipid molecules in MSMS mode delivers subspecies information, i.e., the exact acyl chain (for instance, fatty acid) composition of the lipid molecule. MS-only mode, acquiring data without fragmentation, cannot deliver this information and provides species information only. In that case, the sum of the carbon atoms and double bonds in the hydrocarbon moieties is provided. Lipid species are annotated according to their molecular composition as Lipid class <sum of carbon atoms>:<sum of double bonds>;< sum of hydroxyl groups>. For example, PI 34:1;0 denotes phosphatidylinositol with a total length of its fatty acids equal to 34 carbon atoms, total number of double bonds in its fatty acids equal to 1 and 0 hydroxylations. In case of sphingolipids, SM 34:1;2 denotes an SM species with a total of 34 carbon atoms, 1 double bond, and 2 hydroxyl groups in the ceramide backbone. Lipid subspecies annotation contains additional information on the exact identity of their acyl moieties and their *sn*-position (if available). For example, PI 18:1;0_16:0;0 denotes phosphatidylinositol with octadecenoic (18:1;0) and hexadecanoic (16:0;0) fatty acids, for which the exact position (*sn*-1 or *sn*-2) in relation to the glycerol backbone cannot be discriminated (underline “_” separating the acyl chains). On contrary, PC O-18:1;0/16:0;0 denotes an ether-phosphatidylcholine, where an alkyl chain with 18 carbon atoms and 1 double bond (O-18:1;0) is ether-bound to *sn*-1 position of the glycerol and a hexadecanoic acid (16:0;0) is connect via an ester bond to the *sn*-2 position of the glycerol (slash “/” separating the chains signifies that the *sn*-position on the glycerol can be resolved). Lipid identifiers of the SwissLipids database [48] (http://www.swisslipids.org) are provided in the S4 Table.

### Genomics

#### Genotyping of MDC-CC individuals

Genotyping of Malmö Diet and Cancer Study participants was performed using the Illumina GSA v1 genotyping array. After quality control procedures that removed low-quality samples (discordance between reported and genetically inferred sex, low call rate (<90%), and sample duplicates), 97% of samples were retained. With respect to genetic variants, quality control was performed with removal of those not in Hardy–Weinberg equilibrium (*p* < 1 × 10^−15^). Imputation was performed using a reference panel from the Haplotype Reference Consortium [49].

### Data analysis

Data were analyzed with R version 4.0.4 [50] using tidyverse packages version 1.3.0 [51].

#### Polygenic risk score calculation

Estimates of effect sizes of genomic variants associated with T2D and CVD were taken from the summary statistics of published GWAS [5,6]. These effect sizes were applied on the MDC-CC data to calculate polygenic risk scores (PRS) using PRSice-2 [52]. Briefly, the method selects from a large set of variants the subset which highest predictive power in regard to a given outcome or phenotype (in our case, disease incidences versus controls). The PRS of an individual is then the sum of effect sizes of all variants from this subset that are observed for that person.

#### Predictive risk modeling

In order to compute lipidomic and other composite risk scores, we sought to emulate the approach used for PRS calculation, with few modifications. Our objective was to derive risk scores for all the individuals (3,688 for T2D and 3,951 for CVD). We therefore performed 10 iterations of the modeling (“outer loop”). In each iteration, we randomly split the data into 2/3 training and 1/3 test sets. A model is then trained on the training set and applied to the individuals from the test set. The parameter estimation for a training set involved another 10-fold cross-validation (“inner loop”). A particular individual will be part of the training set in some of the 10 iterations of the outer loop and part of the test set in the other iterations. The final risk score of that individual is then simply the average of all his/her risk scores obtained when this individual was part of a test set, and the associated confidence was assessed via the standard error of the mean (SEM). Similarly, the final effect sizes of predictor variables were computed by averaging over the effect sizes (coefficients) of the 10 individual models.

To account for the high multicollinearity in the lipidomics data, we used classification models that integrate all variables considered for a particular composite risk score into a single analysis. This stands in contrast to PRS calculation where effect sizes are typically estimated separately for each variant. We used elastic net classification for modeling via the R package Caret version 6.0-86 [53] and tested different alpha parameter values from 0 to 1. We did not observe major difference of model performance with respect to alpha and chose alpha = 0 (corresponding to Ridge classification) for the final analysis. Each continuous variable was log-transformed and center-scaled prior to the modeling analysis.

Complementary to the Ridge-based analysis, we also computed risk scores based on Cox proportional hazards models and accounted for time to first incidence during model building. An upfront principal component analysis was done on the joint set of predictor variables of a particular model to account for multicollinearity. We considered as many principal components as there were predictor variables.

The final risk score, either based on a single type of measurement or on a combination of different variable types, is used for ranking of the individuals and stratifying them into equally sized risk subgroups (quantiles). Importantly, since the outcome is known for the test individuals in this study, a quantile may contain both control and future disease cases. The fraction of cases within a quantile as well as the strength of the increase of this case rate with increasing risk score value may thus be used to assess the predictive power of a score.

#### Clustering analysis and statistical comparisons

We computed a hierarchical clustering of the lipidomics data using the *ward*.*D2* method in R. We calculated Pearson correlation coefficients between each lipidome pair, which we substracted from 1 to obtain a distance measure for the clustering. Lipidome-based subgroups were derived by cutting the dendrogram at the height that corresponded to the 6 most basal clusters. We refer to these clusters as 6 subgroups.

Statistical differences of distribution means of various parameters was assessed using Welch’s *t* test (unequal variances *t* test) in case of continuous variables and chi-squared tests in case of discrete values (for instance, sex). Resulting *p*-values were adjusted for multiple testing using the Benjamini–Hochberg (BH) method [54]. Continuous variables (including lipid amounts) were center-scaled prior to the statistical testing, while mean and fold-change values were calculated using the unscaled data. Risk scores were center-scaled prior to the correlation analyses.

#### Publication count

Pubmed counts were retrieved with the R package RISmed [55] (version 2.1.7) using pubmed created Entry Term mappings, for instance, for lipidomics: *“lipidomics”[MeSH Terms] OR “lipidomics”[All Fields] OR “lipidome”[All Fields] OR “lipidomes”[All Fields] OR “lipidomic”[All Fields] AND 2019[dp]’* with the functions EUtilsSummary() and QueryCount().

## Supporting information

S1 Fig Risk prediction improvement by the lipidomic and polygenic features on top of the clinical variables. Comparison of N + C, N + P + C, N + L + C, and N + L + P + C risk scores (A, C) and associated effect sizes of individual predictor variables (B, D) for T2D (top row) and CVD (bottom row). For details, see legend of main Fig 1. The data underlying this figure may be found in S1 Data. BMI, body mass index; CE, cholesteryl ester; Cer, ceramide; Chol, cholesterol; CVD, cardiovascular disease; DAG, diacylglyceride; FBG, fasting blood glucose; HbA1c, glycated hemoglobin; HDL, high-density lipoprotein; LDL, low-density lipoprotein; LPC, lysophosphatidylcholine; LPE, lysophosphatidylethanolamine; PC, phosphatidylcholine; PC O-, ether- phosphatidylcholine; PE, phosphatidylethanolamine; PE O-, ether- phosphatidylethanolamine; PI, phosphatidylinositol; PRS, polygenic risk score; SBP, systolic blood pressure; SM, sphingomyelin; TAG, triacylglyceride; TRIGL, triglyceride; T2D, type 2 diabetes.
(PDF)

S2 Fig Clinical risk score versus time to first incidence. The time in years to the first incidence event is compared to the C risk score for T2D (A) and CVD (B). Pearson’s *r* = −0.299 and −0.23 for T2D and CVD, respectively. The risk scores are average values from 10 independent replications. The curve shows a least squares fit of a linear model to the data. The data underlying this figure may be found in S1 Data. CVD, cardiovascular disease; T2D, type 2 diabetes.
(PDF)

S3 Fig T2D and CVD risk overlap. (A) Effect sizes of the predictor variables considered in the N + L + P + C model are compared between T2D and CVD. Points and bars show, respectively, mean values and SEMs for 10 independent replications. The solid line indicates the diagonal. Values and names of all predictor variables are shown in S1 Table. (B) The T2D risk scores (left) and CVD risk scores (right) are ranked, and those scores within the 90%–100% quantile are connected to the corresponding risk score rank for the other disease by a line. The data underlying this figure may be found in S1 Data. CVD, cardiovascular disease; SEM, standard error of the mean; T2D, type 2 diabetes.
(PDF)

S4 Fig High-risk fatty residue signatures. Shown are fold-changes of fatty residue amounts, summed over all lipid classes, in the individuals of the 90%–100% N + L + P + C risk score quantile relative to those of each of the other 9 quantiles for T2D (A) and CVD (B). Fold-change values of separate quantile comparisons per fatty residue are shown by points, which have high opacity if the difference of mean lipid concentration was statistically significant (after correction for multiple testing) according to a *t* test; otherwise, points are drawn with high transparency for nonsignificant changes. Positive and negative values correspond to, respectively, increased and decreased concentrations in the high-risk 90%–100% quantile participants, while the horizontal line at zero indicates no change. Points corresponding to the same fatty acid are connected by a line. The data underlying this figure may be found in S1 Data. CVD, cardiovascular disease; T2D, type 2 diabetes.
(PDF)

S5 Fig Risk scores under the Cox model. Comparison of N, P, L, N + L + P, and N + L + P + C risk scores for T2D (top) and CVD (bottom) generated using Cox proportional hazards models instead of Ridge classification models that were used in the main analysis (A, D). AUC values for T2D are N: 0.502, P: 0.613, L: 0.716, N + L + P: 0.734, N + L + P + C: 0.789. AUC values for CVD are N: 0.512, P: 0.534, L: 0.616, N + L + P: 0.588, N + L + P + C: 0.600. Relation of the L score with time to first incidence event (B, E) and of the C score with time to first incidence event (C, F) is shown. For details, see legend of Figs 1 and 2. The data underlying this figure may be found in S1 Data. AUC, area under the curve; CVD, cardiovascular disease; T2D, type 2 diabetes.
(PDF)

S1 Table Effect sizes and associated confidence intervals for the parameters of the different model for T2D and CVD. CVD, cardiovascular disease; T2D, type 2 diabetes.
(XLSX)

S2 Table Results of statistical comparisons of lipid species concentrations between the highest 90%–100% risk quantile and other quantiles for T2D (sheet 1) and CVD (sheet 2). CVD, cardiovascular disease; T2D, type 2 diabetes.
(XLSX)

S3 Table Results of statistical comparisons of lipid species and class concentrations and other model parameter values between subgroup 6 and the other subgroups. (XLSX)

S4 Table Mapping of lipid species names used in this study and SwissLipid names and identifiers. (XLSX)

S1 Data Data underlying all main and supporting information figures. (XLSX)

## Abbreviations

AUC: area under the curve
BH: Benjamini–Hochberg
BMI: body mass index
CAD: coronary artery disease
CVD: cardiovascular disease
FBG: fasting blood glucose
GWAS: genome-wide association studies
HbA1c: glycated hemoglobin
HC: hydrocarbon chain
HDL: high-density lipoprotein
LDL: low-density lipoprotein
MDC-CC: Malmö Diet and Cancer-Cardiovascular Study
OR: odds ratio
PRS: polygenic risk score
SBP: systolic blood pressure
SEM: standard error of the mean
SM: sphingomyelin
SNV: single nucleotide variant
T2D: type 2 diabetes
WHO: World Health Organization
