# Supplementary figures and images for: Lipidomic risk scores are independent of polygenic risk scores and can predict incidence of diabetes and cardiovascular disease in a large population cohort
Source: PLoS Biol. 2022 Mar 3;20(3):e3001561. doi: 10.1371/journal.pbio.3001561 (PMC8893343; doi:10.1371/journal.pbio.3001561)

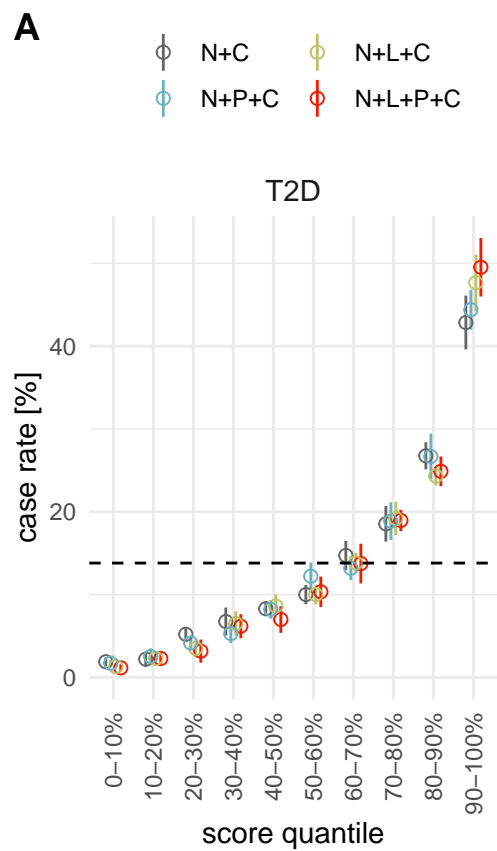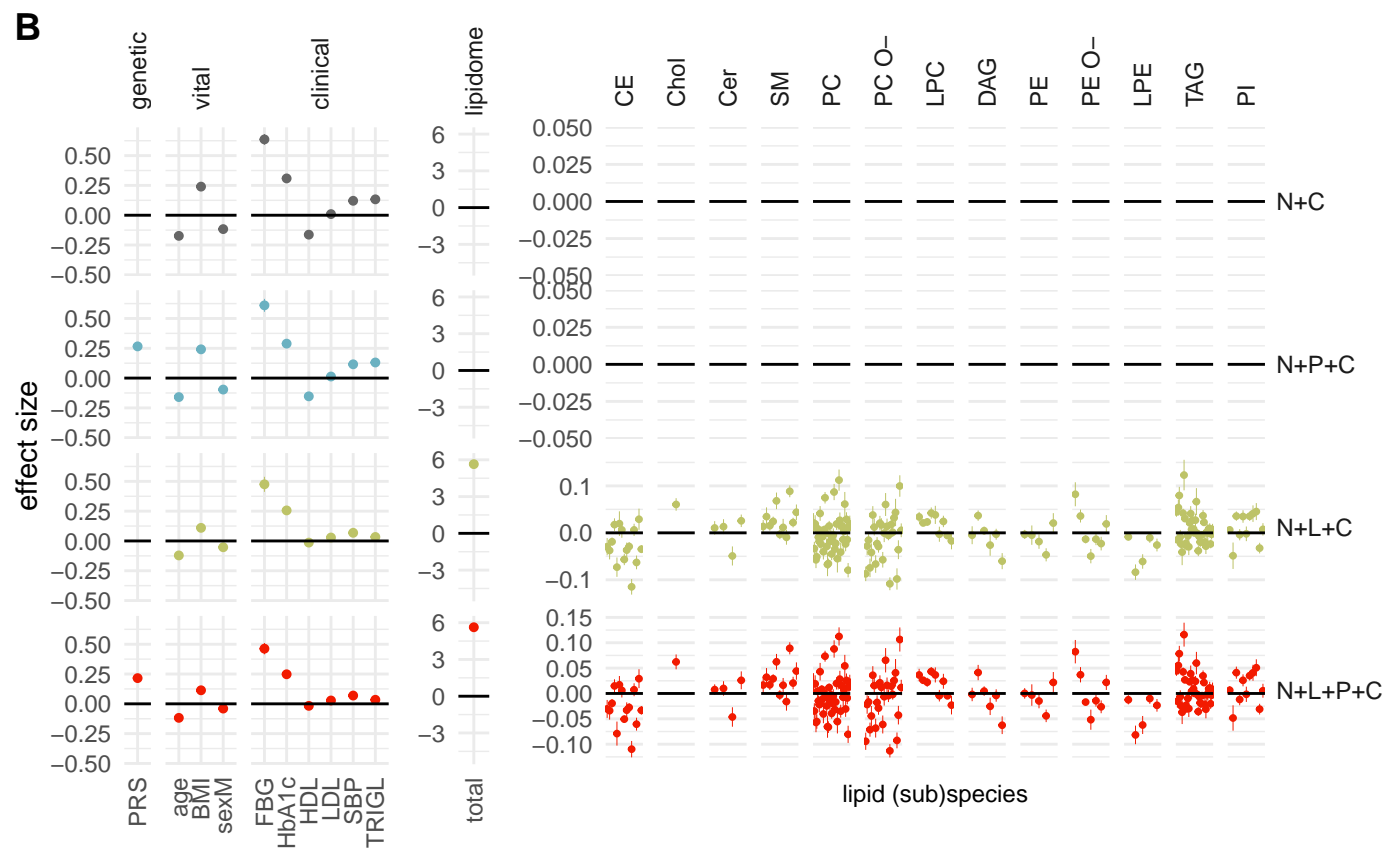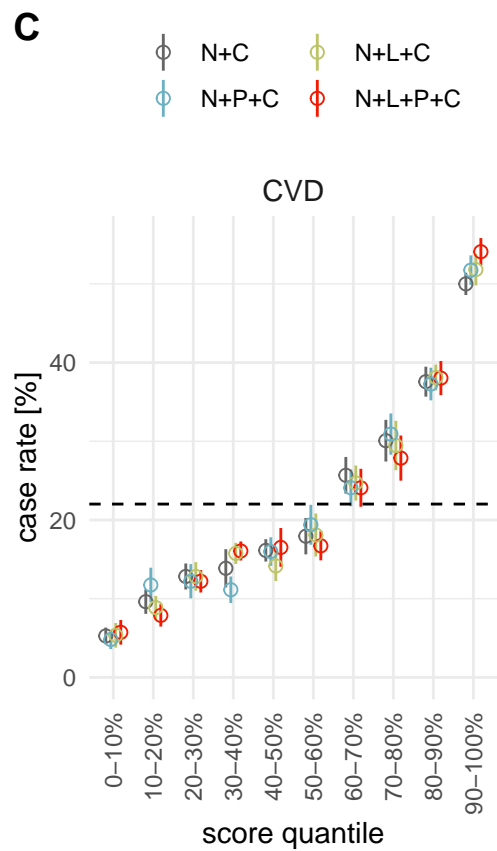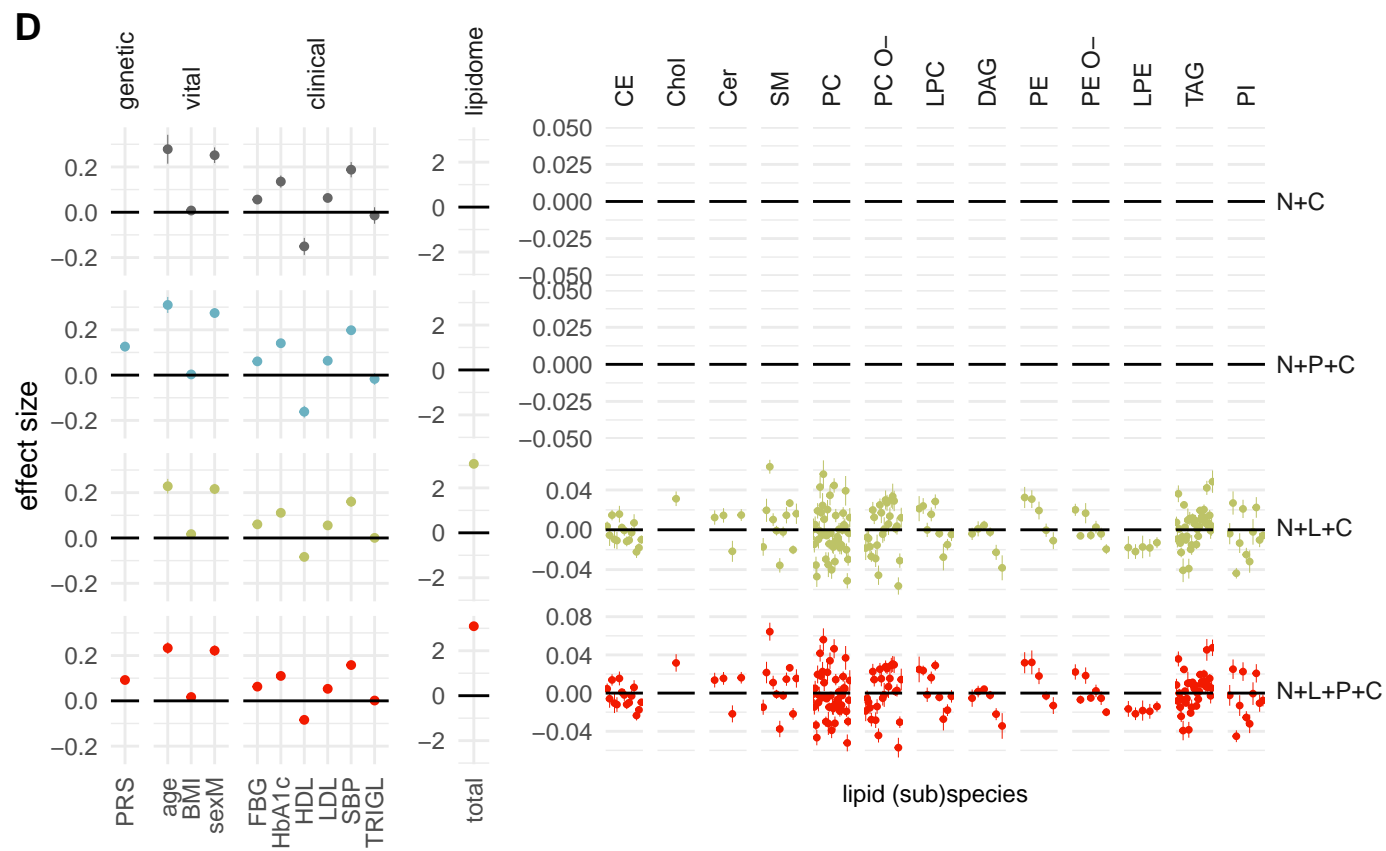

Supplement: S1 Fig — Comparison of N + C, N + P + C, N + L + C, and N + L + P + C risk scores (A, C) and associated effect sizes of individual predictor variables (B, D) for T2D (top row) and CVD (bottom row). For details, see legend of main Fig 1. The data underlying this figure may be found in S1 Data. BMI, body mass index; CE, cholesteryl ester; Cer, ceramide; Chol, cholesterol; CVD, cardiovascular disease; DAG, diacylglyceride; FBG, fasting blood glucose; HbA1c, glycated hemoglobin; HDL, high-density lipoprotein; LDL, low-density lipoprotein; LPC, lysophosphatidylcholine; LPE, lysophosphatidylethanolamine; PC, phosphatidylcholine; PC O-, ether- phosphatidylcholine; PE, phosphatidylethanolamine; PE O-, ether- phosphatidylethanolamine; PI, phosphatidylinositol; PRS, polygenic risk score; SBP, systolic blood pressure; SM, sphingomyelin; TAG, triacylglyceride; TRIGL, triglyceride; T2D, type 2 diabetes. (PDF) [file pbio.3001561.s001.pdf]

**A****T2D**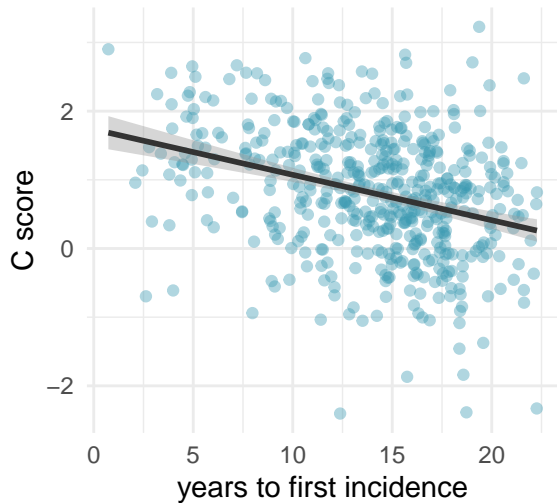**B****CVD**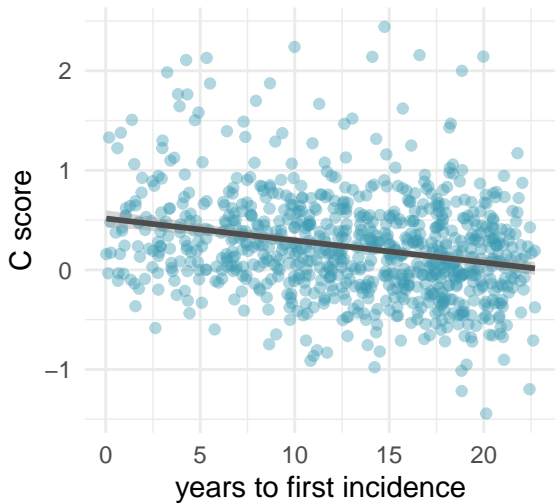

Supplement: S2 Fig — The time in years to the first incidence event is compared to the C risk score for T2D (A) and CVD (B). Pearson’s r = −0.299 and −0.23 for T2D and CVD, respectively. The risk scores are average values from 10 independent replications. The curve shows a least squares fit of a linear model to the data. The data underlying this figure may be found in S1 Data. CVD, cardiovascular disease; T2D, type 2 diabetes. (PDF) [file pbio.3001561.s002.pdf]

**A**

effect size CVD

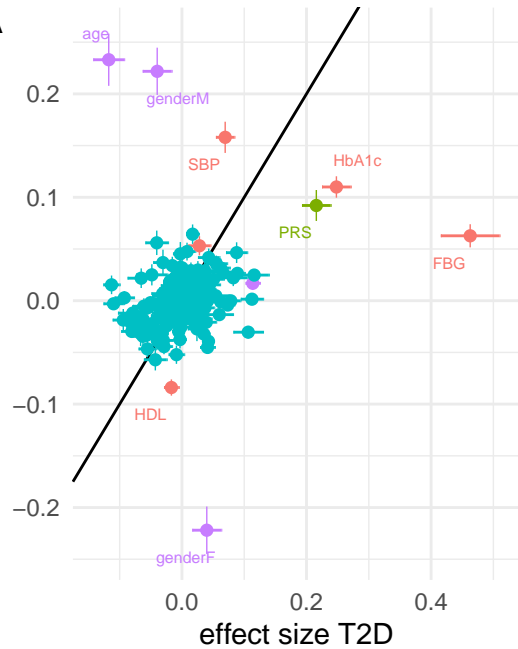**B**

risk score rank

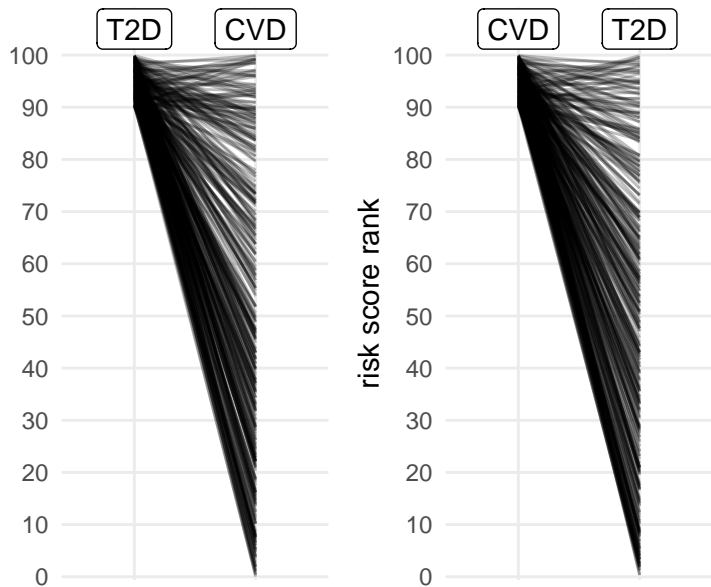

Supplement: S3 Fig — (A) Effect sizes of the predictor variables considered in the N + L + P + C model are compared between T2D and CVD. Points and bars show, respectively, mean values and SEMs for 10 independent replications. The solid line indicates the diagonal. Values and names of all predictor variables are shown in S1 Table. (B) The T2D risk scores (left) and CVD risk scores (right) are ranked, and those scores within the 90%–100% quantile are connected to the corresponding risk score rank for the other disease by a line. The data underlying this figure may be found in S1 Data. CVD, cardiovascular disease; SEM, standard error of the mean; T2D, type 2 diabetes. (PDF) [file pbio.3001561.s003.pdf]

**A****T2D**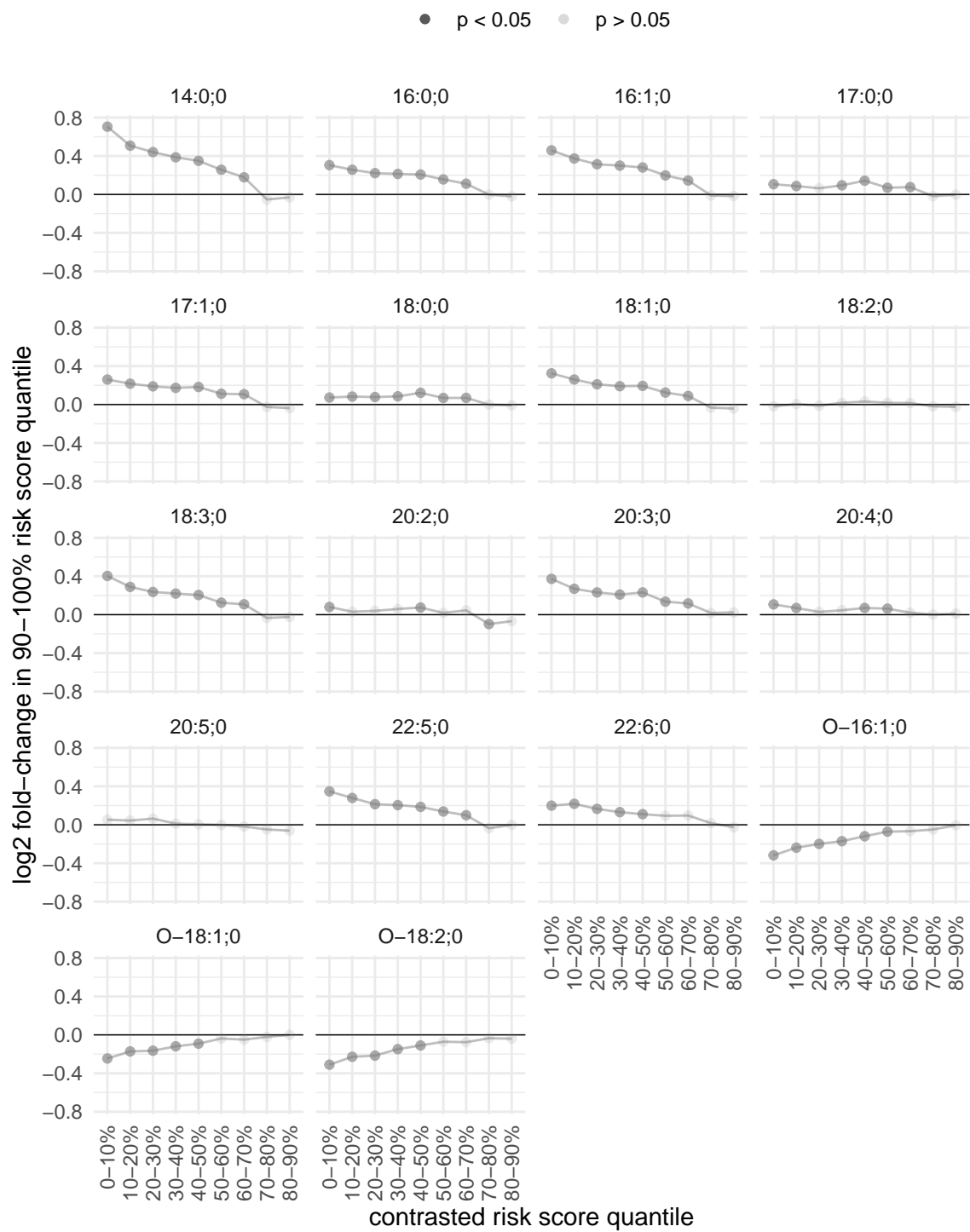**B****CVD**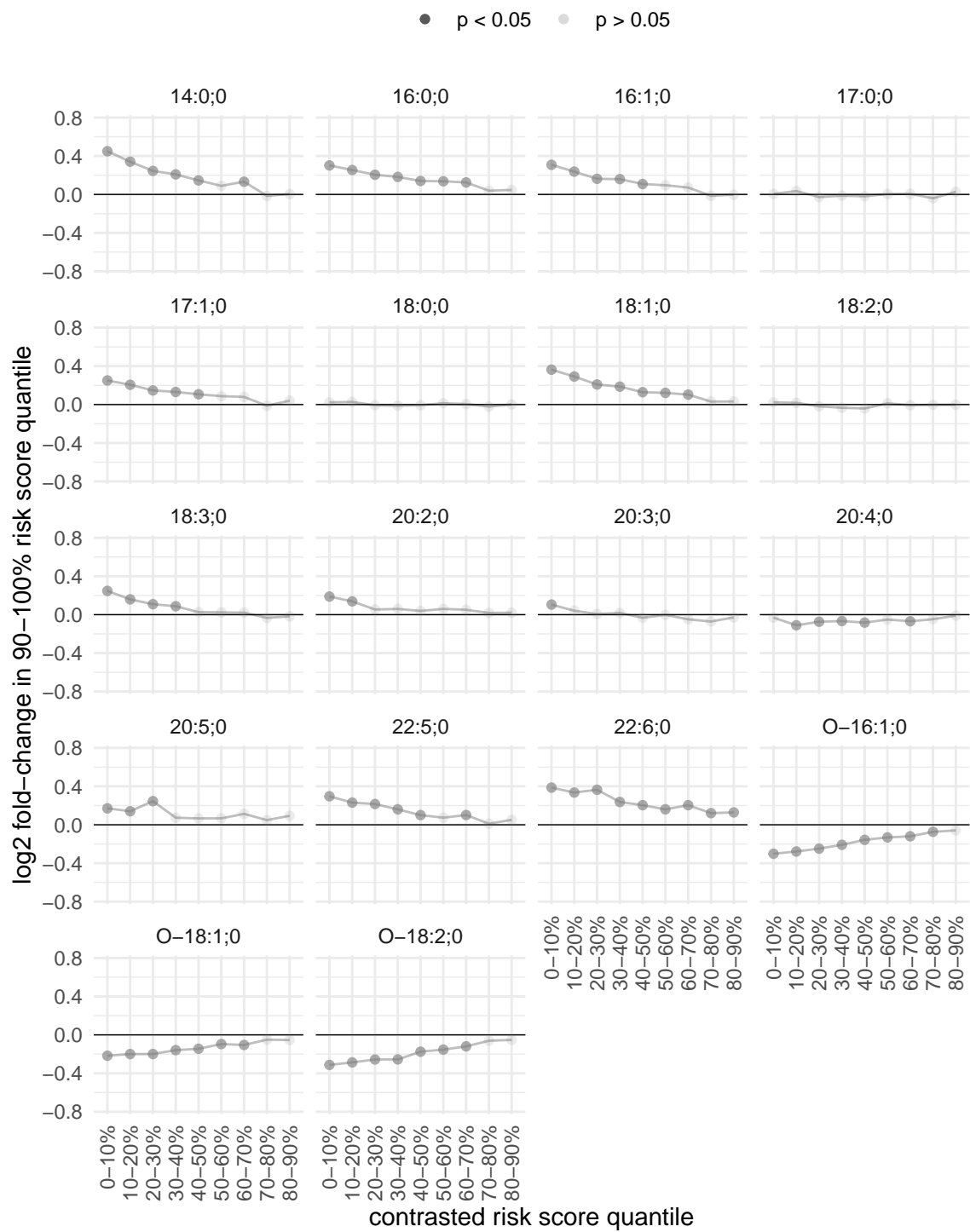

Supplement: S4 Fig — Shown are fold-changes of fatty residue amounts, summed over all lipid classes, in the individuals of the 90%–100% N + L + P + C risk score quantile relative to those of each of the other 9 quantiles for T2D (A) and CVD (B). Fold-change values of separate quantile comparisons per fatty residue are shown by points, which have high opacity if the difference of mean lipid concentration was statistically significant (after correction for multiple testing) according to a t test; otherwise, points are drawn with high transparency for nonsignificant changes. Positive and negative values correspond to, respectively, increased and decreased concentrations in the high-risk 90%–100% quantile participants, while the horizontal line at zero indicates no change. Points corresponding to the same fatty acid are connected by a line. The data underlying this figure may be found in S1 Data. CVD, cardiovascular disease; T2D, type 2 diabetes. (PDF) [file pbio.3001561.s004.pdf]

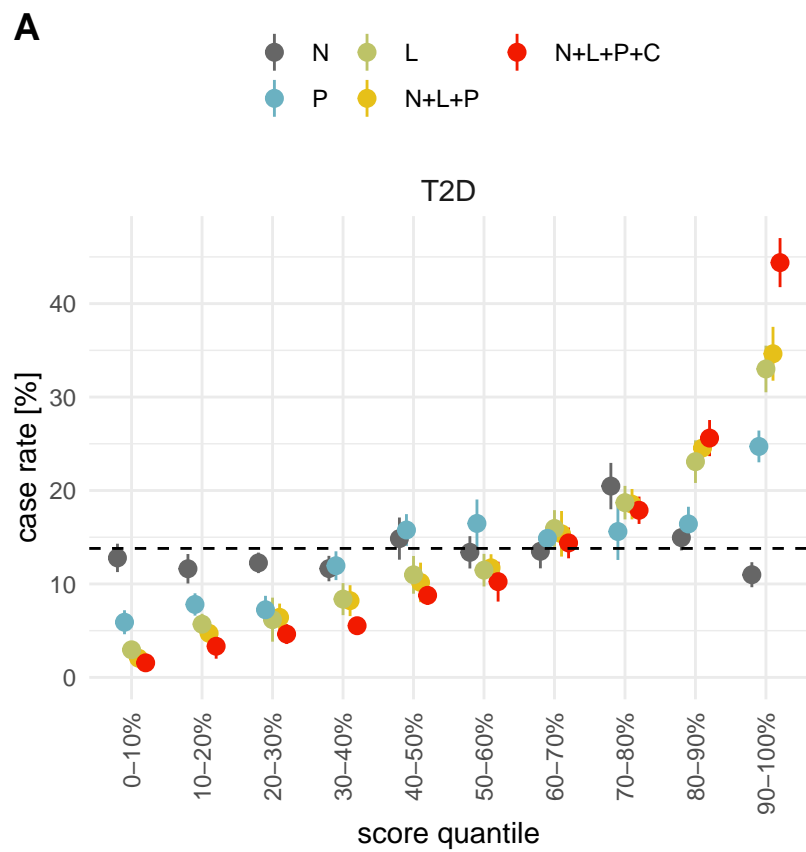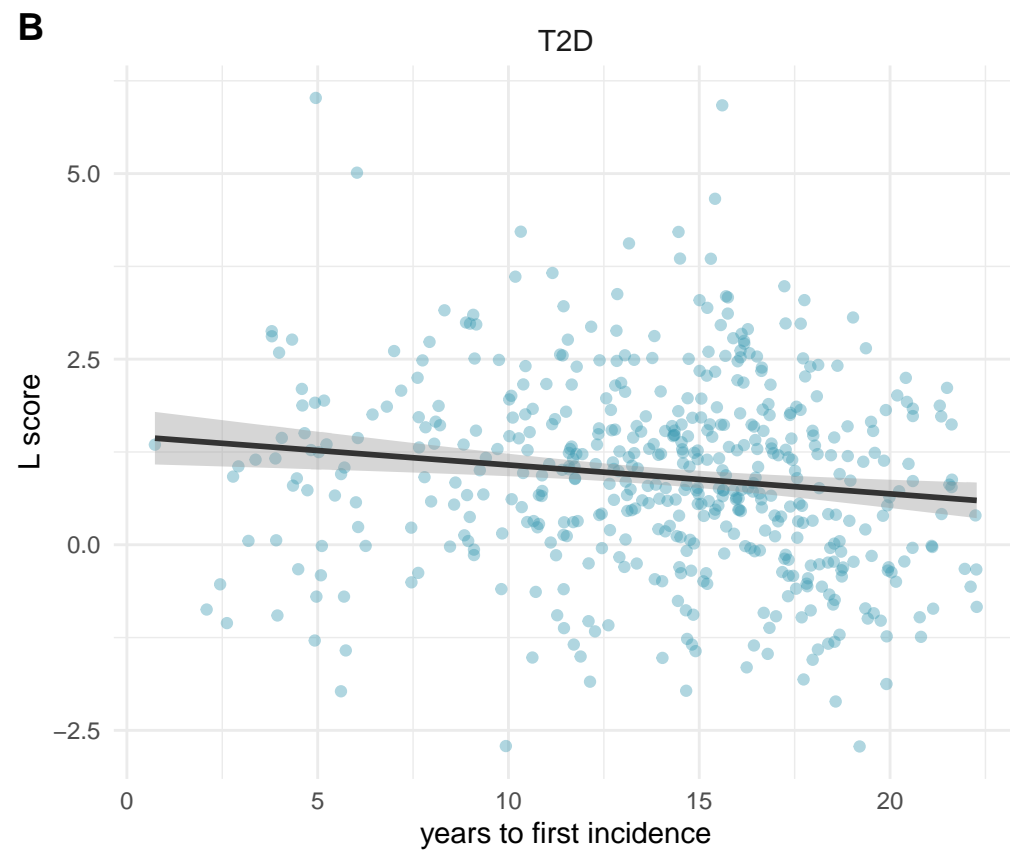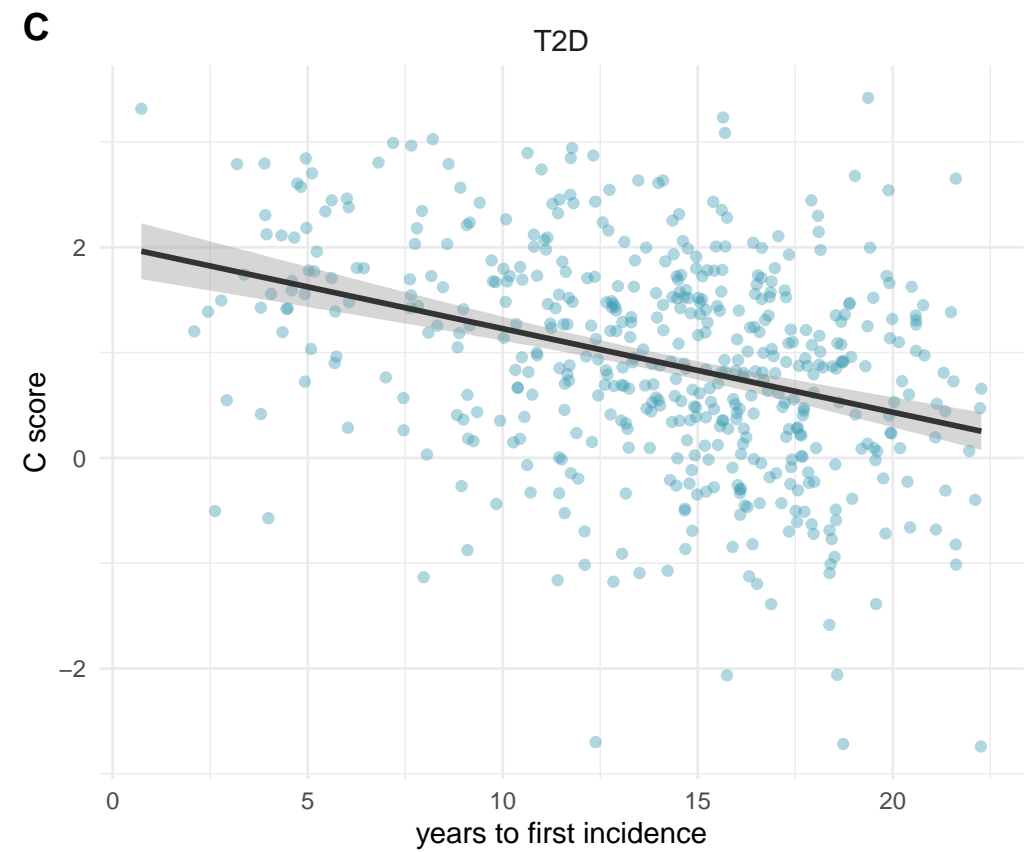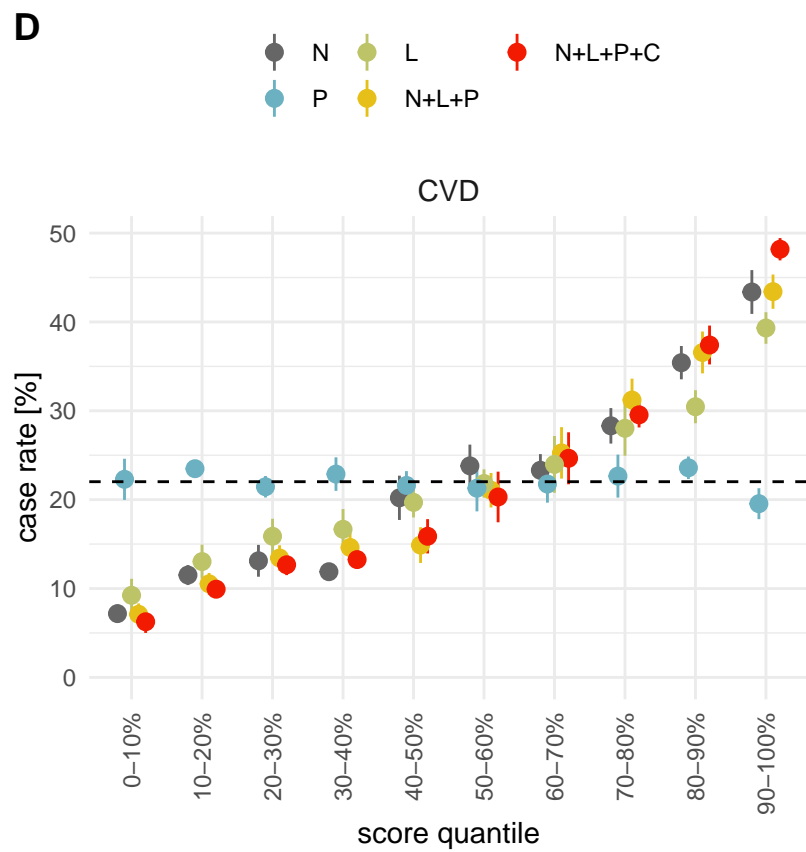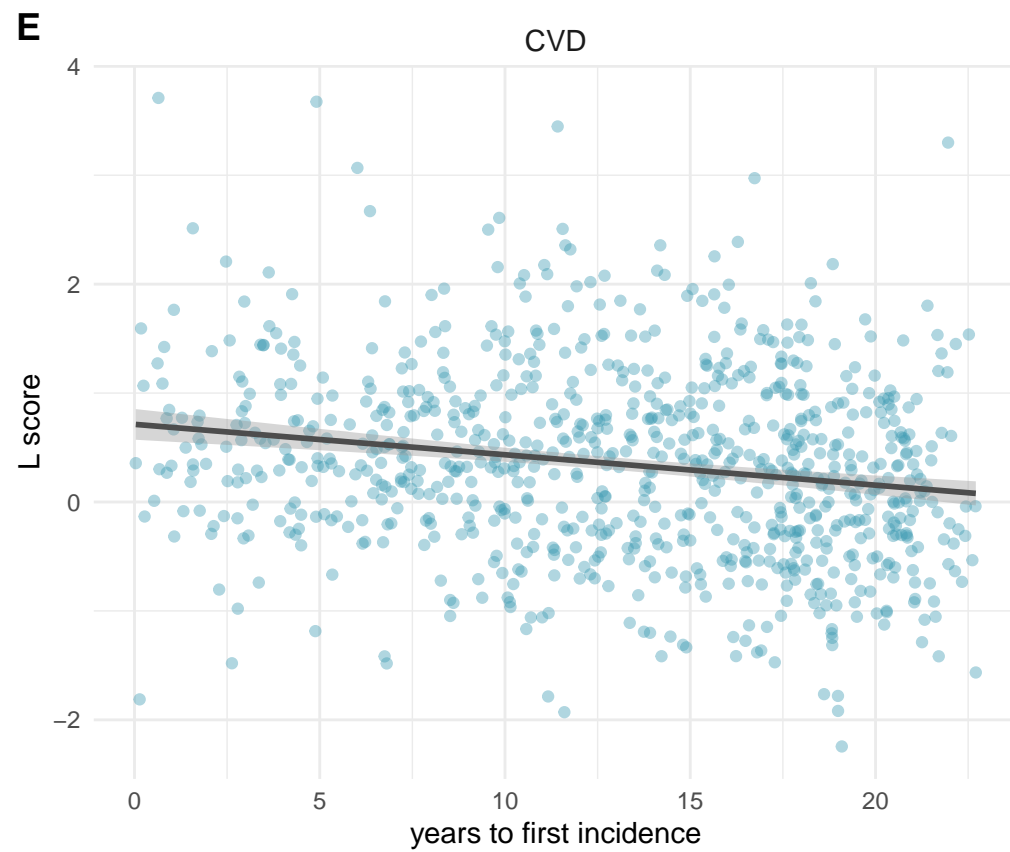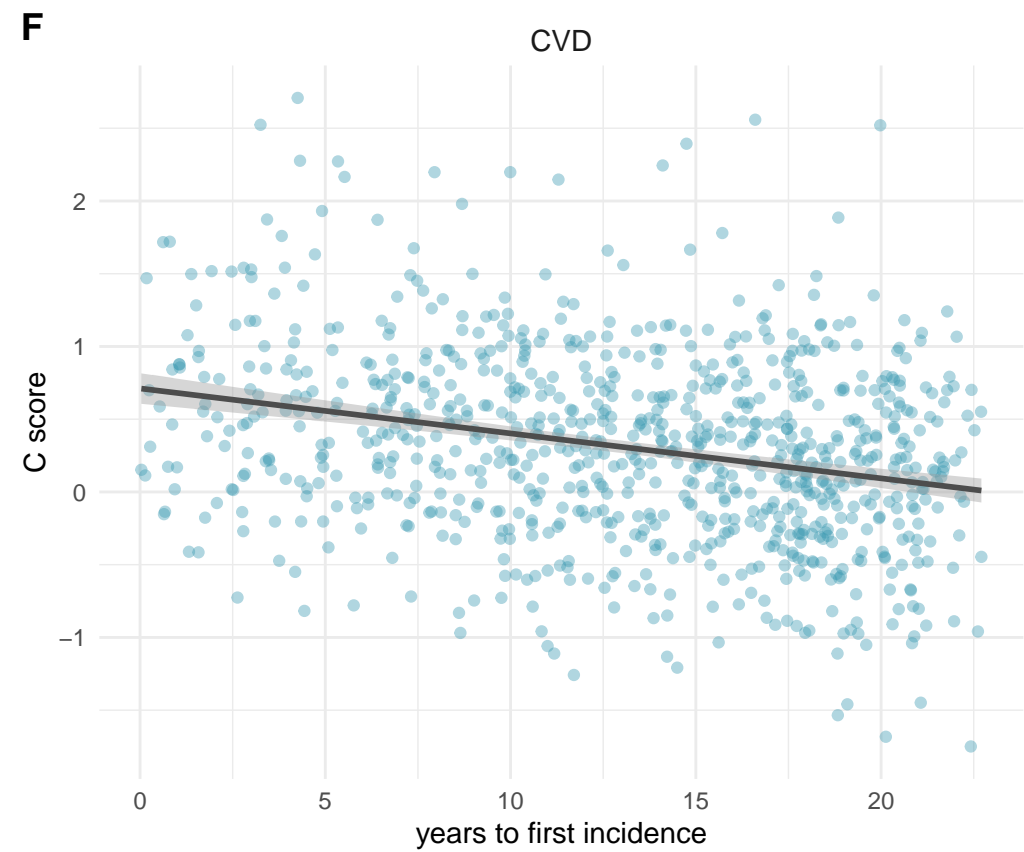

Supplement: S5 Fig — Comparison of N, P, L, N + L + P, and N + L + P + C risk scores for T2D (top) and CVD (bottom) generated using Cox proportional hazards models instead of Ridge classification models that were used in the main analysis (A, D). AUC values for T2D are N: 0.502, P: 0.613, L: 0.716, N + L + P: 0.734, N + L + P + C: 0.789. AUC values for CVD are N: 0.512, P: 0.534, L: 0.616, N + L + P: 0.588, N + L + P + C: 0.600. Relation of the L score with time to first incidence event (B, E) and of the C score with time to first incidence event (C, F) is shown. For details, see legend of Figs 1 and 2. The data underlying this figure may be found in S1 Data. AUC, area under the curve; CVD, cardiovascular disease; T2D, type 2 diabetes. (PDF) [file pbio.3001561.s005.pdf]
